# Silybin A from *Silybum marianum* reprograms lipid metabolism to induce a cell fate-dependent class switch from triglycerides to phospholipids

**DOI:** 10.7150/thno.99562

**Published:** 2025-01-06

**Authors:** Solveigh C. Koeberle, Maria Thürmer, Fengting Su, Markus Werner, Julia Grander, Laura Hofer, André Gollowitzer, Loc Le Xuan, Felix J. Benscheid, Ehsan Bonyadi Rad, Armando Zarrelli, Giovanni Di Fabio, Oliver Werz, Valeria Romanucci, Amelie Lupp, Andreas Koeberle

**Affiliations:** 1 Institute of Pharmaceutical Sciences/Pharmacognosy and Excellence Field BioHealth, University of Graz, 8010 Graz, Austria.; 2 Michael Popp Institute and Center for Molecular Biosciences Innsbruck (CMBI), University of Innsbruck, 6020 Innsbruck, Austria.; 3 Department of Pharmaceutical/Medicinal Chemistry, Institute of Pharmacy, Friedrich Schiller University Jena, 07743 Jena, Germany.; 4 Department of Chemical Sciences, University of Napoli Federico II, I-80126 Naples, Italy.; 5 Institute of Pharmacology and Toxicology, Jena University Hospital, Jena, Germany.

**Keywords:** silybin, liver, lipid metabolism, triglycerides, phospholipids

## Abstract

**Rationale:** *Silybum marianum* is used to protect against degenerative liver damage. The molecular mechanisms of its bioactive component, silybin, remained enigmatic, although membrane-stabilizing properties, modulation of membrane protein function, and metabolic regulation have been discussed for decades.

**Methods**: Experiments were performed with hepatocyte cell lines and primary monocytes *in vitro* under both basal and stressed conditions, and in mice *in vivo*. Quantitative lipidomics was used to detect changes in phospholipids and triglycerides. Key findings were confirmed by Western blotting, quantitative PCR, microscopy, enzyme activity assays, metabolic flux studies, and functional relationships were investigated using selective inhibitors.

**Results**: We show that specifically the stereoisomer silybin A decreases triglyceride levels and lipid droplet content, while enriching major phospholipid classes and maintaining a homeostatic phospholipid composition in human hepatocytes *in vitro* and in mouse liver *in vivo* under normal and pre-disease conditions. Conversely, in cell-based disease models of lipid overload and lipotoxic stress, silybin treatment primarily depletes triglycerides. Mechanistically, silymarin/silybin suppresses phospholipid-degrading enzymes, induces phospholipid biosynthesis to varying degrees depending on the conditions, and down-regulates triglyceride remodeling/biosynthesis, while inducing complex changes in sterol and fatty acid metabolism. Structure-activity relationship studies highlight the importance of the 1,4-benzodioxane ring configuration of silybin A in triglyceride reduction and the saturated 2,3-bond of the flavanonol moiety in phospholipid accumulation. Enrichment of hepatic phospholipids and intracellular membrane expansion are associated with a heightened biotransformation capacity.

**Conclusion**: Our study deciphers the structural features of silybin contributing to hepatic lipid remodeling and suggests that silymarin/silybin protects the liver in individuals with mild metabolic dysregulation, involving a lipid class switch from triglycerides to phospholipids, whereas it may be less effective in disease states associated with severe metabolic dysregulation.

## Introduction

Hepatic pathologies such as metabolic dysfunction-associated steatotic liver disease (MAFLD; former: non-alcoholic fatty liver disease, NAFLD 1), metabolic dysfunction-associated steatohepatitis (MASH; former: non-alcoholic steatohepatitis, NASH), fibrosis, and cirrhosis are closely related to the metabolic syndrome and insulin resistance 2-6. They are driven by high-calorie diets that induce abnormal glucose and lipid metabolism and subsequently cause glucotoxicity, lipotoxicity, oxidative stress, and chronic inflammation 2,7-9. As a consequence, fatty acids are taken up by hepatocytes, and also synthesized *de novo* 10*,* incorporated into triglycerides (TGs), and stored in lipid droplets 11-13. While the transfer of fatty acids into lipid droplets contributes to the detoxification of excess free fatty acids 13, a chronic increase in the number and size of lipid droplets induces hepatocyte enlargement and dysfunction 7,14. This continuous lipid accumulation leads to hepatic steatosis and, as the disease progresses, to cirrhosis and hepatocellular carcinoma 2,15. As an adaptive strategy to protect hepatocytes from lipid overload, autophagy of lipid droplets (lipophagy) is initiated 16 and the mobilized fatty acids are subjected to oxidative degradation 17. Compensatory upregulation of fatty acid oxidation at the onset of MAFLD provides partial relief but is insufficient to reduce hepatic lipids to basal levels. In addition, the increased oxidative breakdown of lipids induces oxidative stress, which can negatively contribute to cell and tissue damage 7,18. MAFLD is also significantly influenced by genetic factors 19. Candidate gene variants act in multiple pathways of lipid metabolism 20, including *de novo* lipogenesis and lipid droplet assembly (LPIN2, ATGL/PNPLA2)^21^,^22^, phospholipid biosynthesis and remodeling (LPIAT1/MBOAT7, iPLA2/PLA2G6, PNPLA8, PRDX6, PLD1)^23^-^29^, neutral and phospholipid hydrolysis and catabolism (PNPLA3)^30^, sterol metabolism (HSD17B13)^31^ fatty acid compartmentalization (GCKR, TM6SF2), and lipoprotein assembly and secretion (PLA2G7, TM6SF2)^26^. Consequently, both MAFLD and MASH are characterized by extensive changes in hepatic lipid composition, including a decrease in total phosphatidylcholine (PC) and an increase in TG 32-35.

**Figure 1:**
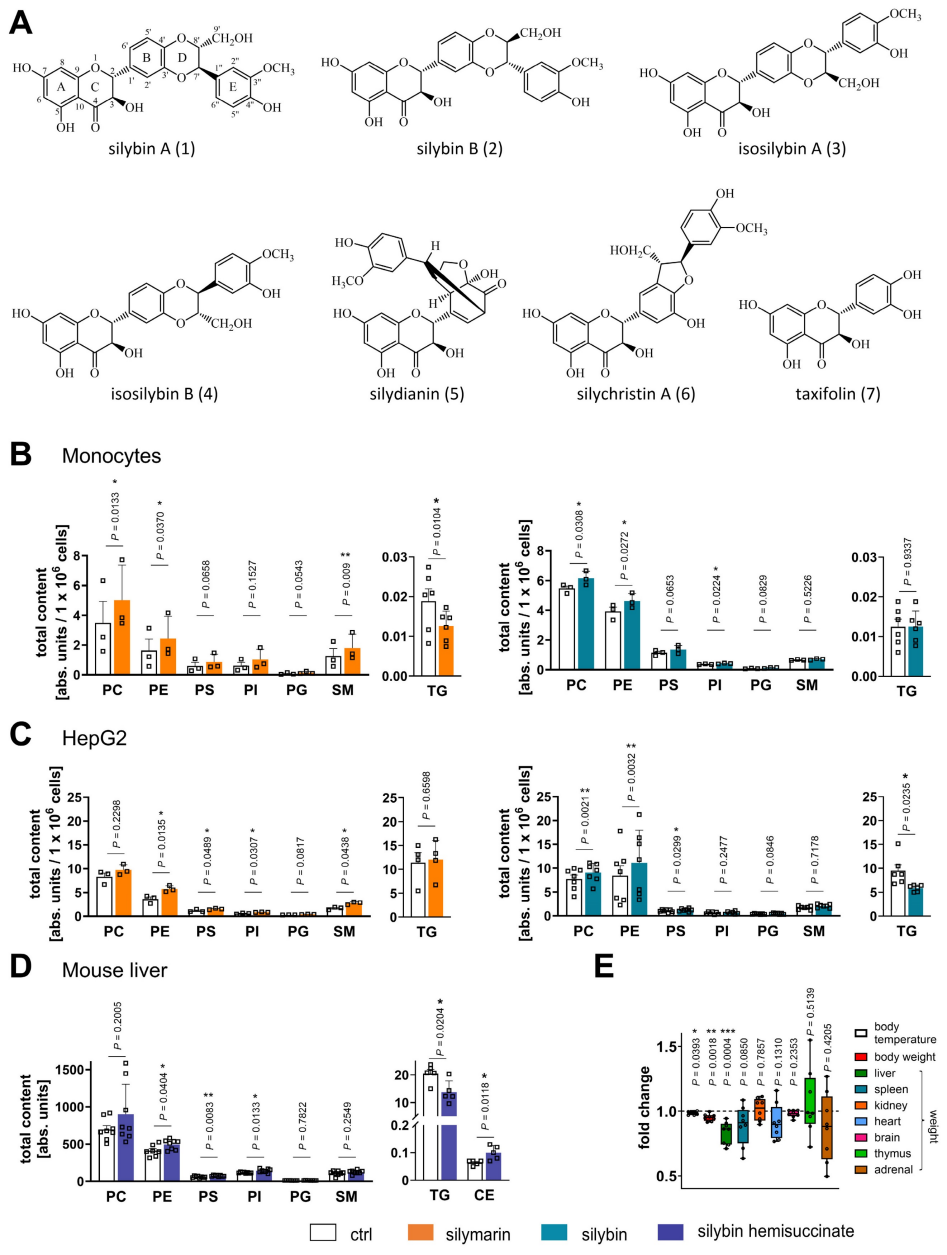
Shift from TGs to phospholipids in human monocytes, hepatocytes, and mouse liver. (A) Main components of silymarin. (B-D) Total amounts of lipid classes were determined by UPLC-MS/MS. (B, C) Human primary monocytes (B) and HepG2 cells (C) were treated with silymarin (50 µg/ml for monocytes and 10 µg/ml for HepG2 cells), silybin (20 µM), or vehicle (ethanol for silymarin, DMSO for silybin) for 24 h. Individual values and mean + SEM; n = 3 (B: except TG, C: silymarin except TG), n = 4 (C: TG silymarin), n = 6 (B: TG silymarin and silybin, C: TG silybin), n = 7 (C: silybin except TG). (D, E) Mice received silybin hemisuccinate ('silybin'; 200 mg/kg, i.p.) or vehicle (0.9% NaCl) trice at 0, 12, and 24 h and were sacrificed after 37 h. (E) Body temperature, body weight and organ weight of mice upon administration of silybin. Temperature and body weight were measured after 37 h before animals were sacrificed and organs collected. The box-and-whisker plot shows fold-changes upon silybin gavage. The median fold change belonging to each group is shown as bold line. The boxes extend from the 25^th^ to 75^th^ percentiles, and whiskers extend to minimal and maximal values. Vehicle control; body temperature: 37.2 ± 0.2 [°C]; body weight: 21.9 ± 0.2 [g]; liver: 1.31 ± 0.03 [g]; spleen: 0.070 ± 0.002 [g]; kidney: 0.1445 ± 0.004 [g]; heart: 0.126 ± 0.0023 [g]; brain: 0.439 ± 0.006 [g]; thymus: 0.049 ± 0.002 [g]; adrenal: 0.0105 ± 0.001 [g]; Lipid contents are given as nmol / 1×10^6^ cells for PC and units / 1×10^6^ cells for PE, PS, PI, PG, SM and TG. Individual values and mean + SEM (D) or box plots and individual values (E) from n = 5 (D: CE and TG), n = 7 (D: PE; ctrl, E: body temperature), n = 8 (D: except CE and TG, E: body and organ weights) mice/group. *P < 0.05, **P < 0.01, ***P < 0.001 vs. vehicle control. Two-tailed paired (B, C) or unpaired (D, E) Student's *t*-test.

Milk thistle (*Silybum marianum* L.) is a medicinal plant that is traditionally used for the treatment of liver and biliary tract diseases 36-39 and a variety of other pathologies, including diabetes 40 and cancer 41,42. Organic fruit extracts (silymarin) of *S. marianum* consist of the flavonolignans silybin A and B (~30%), isosilybin (~5%), silychristin A (~7%), and silydianin (~10%), the flavonoid (+)-taxifolin (~5%) (Figure 1A), and less defined polyphenols (30%) 41,43. Minor constituents include silychristin B, isosilychristin, 2,3-dehydrosilybin, quercetin, and kaempferol 41,43,44. The major biologically active flavonolignan, silybin, also termed as silibinin, exists as a mixture of the two diastereomers silybin A and B 43. Human and animal studies with silymarin or its main component silybin on liver pathologies such as oxidative or lipotoxic stress-induced alcoholic and non-alcoholic fatty liver disease and steatohepatitis show (pre)clinical efficacy 45-49, whereas studies on xenobiotic-induced liver toxicity produced mixed results 36,38,50, with only rare cases of side effects 51. Note that the oral bioavailability of silybin can be substantially boosted by specific formulations, yielding systemic silybin plasma concentrations (C_max_) up to 85 µM in humans 36. The hepatoprotective activities of silymarin/silybin have been ascribed to antioxidant response inducing, anti-inflammatory 52, antifibrotic, hepatocyte regeneration-stimulating, and membrane-stabilizing properties 47,53. Several studies have found that administration of silymarin/silybin reduces levels of low-density lipoprotein (LDL), VLDL, cholesterol, and/or TGs, while other studies have not observed substantial changes in the serum lipid profile 54-59, which is not readily understood but may be related to the dose. Recently, silymarin (but not silybin) has been proposed to decrease lipid accumulation during a high-fat diet by altering the vitamin B12-producing capacity of the gut microbiota 60. On the other hand, silymarin/silybin has been suggested to increase PC biosynthesis by upregulating choline phosphate cytidylyltransferase 61. Silymarin/silybin compensated for the decrease of phosphatidylcholine (PC) and phosphatidylethanolamine (PE) in rat liver upon intoxication 62,63 and, when given as a silybin- and PC-based food integrator to MASH patients, restored plasma PC and sphingomyelin (SM) levels 54. Whether silymarin/silybin actively promotes phospholipid enrichment or indirectly increases phospholipid levels by alleviating disease conditions is insufficiently understood, as are the consequences for other membrane phospholipid classes and the knowledge of phospholipidomic profiles. The latter is of great importance because imbalances in the membrane phospholipid composition can cause severe alterations in membrane architecture and function 64.

Here, we demonstrate that silymarin/silybin increases the levels of phospholipids by suppressing their degradation. This effect is partially combined with the induction of phospholipid biosynthetic enzymes, depending on the condition. Simultaneously, it reduces TG levels by downregulating multiple biosynthetic enzymes or by altering TG remodeling processes in hepatocytes, depending on the specific context. To some extent, this effect is also observed in extrahepatic cell types. We ascribe this activity to specific structural features of silybin A and find that they prevail in healthy or pre-disease states not yet afflicted with massive lipid overload, whereas TG-lowering mechanisms predominate under the latter severe liver disease conditions. The channeling of fatty acids from triglycerides to phospholipids has the advantage of i) reducing hepatic TG levels and lipid droplet size ii) avoiding high lipotoxic levels of free fatty acids, and iii) expanding intracellular membranes, which may explain the enhanced hepatic biotransformation capacity upon treatment with silybin. Major adverse changes in membrane function are not expected from the balanced upregulation of phospholipid species. Conclusively, our data suggest that the mechanism of silymarin/silybin described here is more effective in protecting against metabolic liver disease rather than reversing advanced disease states.

## Materials and Methods

### Materials

Silybin, staurosporine, and atglistatin were obtained from Merck (Darmstadt, Germany), silybin-C-2',3-bis(hydrogen succinate) disodium salt (Legalon^®^ SIL) was from Madaus GmbH (Köln, Germany), the PPARγ antagonist GW9662, and the DGAT1 inhibitor A-922500 were purchased from Cayman Chemicals (Ann Arbor, MI), the DGAT2 inhibitor PF-06424439 was bought from Bio-Techne (Abingdon, United Kingdom), thapsigargin was from Enzo Life Sciences (Farmingdale, NY), and silymarin (Silimarit®) was a kind gift from Bionorica SE (Neumarkt, Germany). Silybin, its derivatives and other compounds were dissolved in DMSO, stored in the dark at ‑20°C under argon, and freezing/thawing cycles were kept to a minimum. Silymarin was freshly dissolved in ethanol at the day of experiment. Phospholipid standards were purchased from Otto Nordwald GmbH (Hamburg, Germany) or Merck Millipore (Darmstadt, Germany), were dissolved in chloroform, aliquoted and stored under argon protected from light at ‑80°C. BODIPY 493/503 and ProLong^TM^ Diamond Antifade Mountant with DAPI were purchased from Thermo Fisher Scientific (Waltham, MA). Rabbit anti-β-actin (13E5; #4970), mouse anti-β-actin (8H10D10; #3700), rabbit anti-acetyl-CoA carboxylase (C83B10; #3676), rabbit anti-ATF-6 (D4Z8V, #65880), rabbit anti-ATGL (#2138), rabbit anti-BiP (C50B12, #3177), rabbit anti-phospho-acetyl-CoA carboxylase (Ser79; D7D11; #11818), rabbit anti-GAPDH (D16H11; #5174), mouse anti-GAPDH (D4C6R; #97166), rabbit anti-FAS (#3189), and rabbit anti-XBP-1s (D2C1F, #12782S) were obtained from Cell Signaling (Danvers, MA). Mouse anti-calnexin (C8.B6; #MAB3126) was from Merck Millipore (Darmstadt, Germany) and mouse anti-GM130 (#610822) from BD Bioscience (San Jose, CA, USA). Goat anti-rat CYP1A1 (#219207), goat anti-rat CYP3A2, (#210167), and goat anti-rat CYP2B1, (#219207) were obtained from Daiichi Pure Chemicals Co. LTD (Tokyo, Japan). Rabbit anti-DGAT1 (NB110-41487SS) and rabbit anti-DGAT2 (NBP1-71701SS) were from Novus Biologicals (Abingdon, UK). Mouse anti-GRP78/BiP (A-10, #sc-376768) was purchased from Santa Cruz Biotechnology (Dallas, TX). Alexa Fluor 555 goat anti-mouse IgG (H+L) and Alexa Fluor 488 goat anti-rabbit IgG (H+L) were purchased from Life Technologies (MA, USA). Secondary antibodies for Western blot studies were from LI-COR Biosciences (Bad-Homburg, Germany) and Thermo Fisher Scientific. Peroxidase-conjugated avidin and the secondary biotinylated antibodies rabbit anti-mouse IgG and rabbit fblanti-goat used in immunohistochemical studies were from VECTASTAIN^®^ Elite ABC-Kit (Vector Laboratories, Burlingame, CA).

### Synthesis of silybin derivatives

Silybin A and B were separated from the diastereomeric mixture silybin (Merck) by preparative HPLC as described 65. Starting from the purified silybin A and B, the two enantiomers of 2,3-dehydrosilybin (A and B) were synthesized in good yields and optically pure by base-catalyzed oxidation under microwave heating 66. The hemiacetal **11**, was obtained in good yield by the microwave conversion of silybin in pyridine at 110°C 66. All products were fully characterized by NMR (^1^H, ^13^C), CD, [α]_D_, and ESI MS analyses. The purities of the products were higher than 98%.

### Cell culture, primary monocytes and cell treatment

Cultured cell lines: Human HepG2 liver carcinoma cells (1×10^5^ cells/cm^2^_,_ Leibniz Institute DSMZ-German Collection of Microorganisms and Cell Cultures, Braunschweig, Germany) were grown in RPMI 1640 medium containing 10% heat-inactivated fetal calf serum (FCS, GE Healthcare, Freiburg, Germany or Merck) at 37°C and 5% CO_2_. Human HepaRG hepatoma cells (1.5-2×10^5^ cells/cm^2^, Biopredic International, Rennes, France) were cultured in Williamˈs E medium (Merck) supplemented with 10% heat-inactivated FCS, 2 mM *L*-glutamine (Merck), 5 μg/ml human insulin (Merck), and 50 μM hydrocortisone (Cayman) at 37°C and 5% CO_2_. Human Caco-2 colorectal adenocarcinoma cells (1.7×10^5^ cells/cm^2^) were cultured in DMEM medium (Merck) containing 10% FCS at 37°C and 5% CO_2_. Cells were detached by trypsin/EDTA and reseeded every 3-4 days before reaching confluence. HepG2 cells were used up to passage 28 and HepaRG cells up to passage 44.

Primary cells: Collection of venous blood in heparinized tubes (16 I.E. heparin/mL blood) was performed by the Institute for Transfusion Medicine of the University Hospital Jena (Germany) with informed consent of registered male and female healthy adult volunteers (18 to 65 years). Blood donors were fasted for at least 12 h, had not taken antibiotics or anti-inflammatory drugs prior to blood donation (> 10 days), and were free of apparent infections, inflammatory disorders, or acute allergic reactions. The volunteers regularly donated blood (every 8 to 12 weeks) and were physically inspected by a clinician. Leukocyte concentrates were prepared, erythrocytes removed by dextran sedimentation, and peripheral blood mononuclear cells (PBMC) were isolated by density gradient centrifugation on lymphocyte separation medium (LSM 1077, GE Healthcare) as previously described 67. The fraction of PBMC was cultivated in RPMI 1640 medium containing 10% FCS in 12-well plates (37°C, 5% CO_2_) at a density of 2×10^7^/ml for 1 to 1.5 h to separate adherent monocytes. The cell population used for further studies consisted of more than 85% monocytes according to forward and side scatter properties and CD14 surface expression (BD FACS Calibur flow cytometer, BD Biosciences, Heidelberg, Germany). Experiments were approved by the ethical commission of the Friedrich-Schiller-University Jena.

Cell treatment: HepG2 cells (1×10^5^ cells/cm^2^) and monocytes (6×10^5^/cm^2^) were seeded and directly exposed to vehicle (0.1% DMSO or 0.05% ethanol), silymarin (50 µg/ml for monocytes and 10 µg/ml for HepG2 cells), silybin A/B (20 µM), or STS (1 µM). Adherent cells were harvested with trypsin/EDTA (Merck or Promega, Madison, WI). For lipid droplet staining with Oil Red O, HepG2 cells were instead seeded in 96-well plates at 20,000 cells per well and incubated for 24 h before treatment with vehicle (0.5% DMSO or 0.5% ethanol), silymarin (10 µg/ml), or silybin A/B (20 μM) for an additional 24 h. Treatment of HepaRG cells is described in section “*Cell-based models of MAFLD and lipotoxic stress*”. For transcriptome analysis, Caco-2 cells (1.7×10^5^ cells/cm^2^) were seeded and directly exposed to vehicle (0.5% DMSO), silymarin (30 µg/ml), and silybin (30 µM) for 24 h. Adherent cells were harvested with trypsin/EDTA.

### Complexation of fatty acids to BSA

BSA (1%, Carl Roth, Karlsruhe, Germany) was dissolved in Williams E medium, sterile filtered (Rotilabo^®^-syringe filter, PVDF, 0.22 µm, Carl Roth), mixed with PA (50 mM) or OA (50 mM), sonicated at 60°C for 30 min using a USC100TH sonicator (VWR, Vienna, Austria, 60 W, 45 kHz), and stored at -20°C. Solutions were mixed vigorously immediately before use.

### Cell-based models of MAFLD and lipotoxic stress

HepaRG cells (10,000 / well, 96-well plate) or 2.5×10^6^ cells/25 cm^2^ were cultured at 37°C and 5% CO_2_ for 24 h. The cell culture medium was replaced with fresh medium supplemented with i) vehicle (1% BSA in Williams E medium), ii) BSA-complexed PA/16:0 (0.1 mM, Merck) and OA/18:1 (Cayman) in a 1:2 ratio (in total 1 mM) to induce massive lipid accumulation (mimicking MAFLD), or iii) BSA-complexed PA (0.1 mM) to induce lipotoxic stress. For lipidomic analysis, cells were either co-treated directly with vehicle (DMSO, 0.5%) or silybin A (20 μM), and the incubation was prolonged for another 24 h. Alternatively, treatment was started 24 h after fatty acid challenge and incubation was prolonged for a further 24 h. For lipid droplet analysis, cells were co-treated with vehicle (DMSO, 0.5%), silybin A (20 μM), the ATGL inhibitor atglistatin (50 µM), the DGAT1 inhibitor A 922500 (5 µM), the DGAT2 inhibitor PF-06424439 (10 µM), a combination of DGAT1 (5 µM) and DGAT2 inhibitors (10 µM), or the PPARγ antagonist GW9662 (5 µM) and the incubation was prolonged for another 24 h or 48 h, respectively. Lipid droplet signals, the number of viable cells and membrane integrity, cellular metabolic activity, and phospholipid and TG levels were determined as described in the respective sections.

### Quantitation of lipid droplets in hepatocytes

HepaRG cells were washed twice with 100 μl PBS pH 7.4 and fixed with paraformaldehyde solution (4% in PBS pH 7.4, Merck) for 40 min at room temperature. After removal of the fixative, the cells were washed twice with 100 μl of water, incubated with aqueous isopropanol (60%, 100 μl, 5 min) to remove polar lipids and reduce background signals, and stained with Oil Red O solution (50 μl) for 25 min at room temperature. The latter was prepared by diluting 0.5% Oil Red O in isopropanol (Merck) 1.7-fold in water, sterile-filtered (Rotilabo^®^-syringe filter, PVDF, 0.22 µm, Carl Roth), and allowed to stand for 10 min before staining. Cells were washed three times with water, and microscopic images were taken using a 40× objective (Motic, Barcelona, Spain) on a Motic AE31E microscope (Motic) equipped with a Motic camera. Alternatively, lipid droplets in HepG2 cells were stained with BODIPY 493/503 and manually counted as described in section “Immunofluorescence microscopy”. For photometric quantitation of the stained lipid droplets, Oil Red O was extracted with 60% isopropanol in water (100 μl) for 10 min at room temperature, and the absorbance of the extracted solution was measured at 510 nm using a multi-mode microplate reader (SpectraMax iD3, Molecular Devices).

### Cell number, viability, morphology, and cell diameter

Cell number, cell viability, and cell diameters were determined after trypan blue staining using a Vi-CELL Series Cell Counter (Beckmann Coulter GmbH, Krefeld, DE). Morphological analysis of the cells was carried out on an Axiovert 200 M microscope with a Plan Neofluar × 100/1.30 Oil (DIC III) objective (Carl Zeiss, Jena, Germany). Images were obtained using an AxioCam MR3 camera (Carl Zeiss).

### Cell viability based on cellular dehydrogenase activity

Cytotoxic effects of silymarin and silybin were determined as described 68. Briefly, HepG2 cells (1×10^5^/well of a 96-well plate) or HepaRG cells were cultured as described in sections “Cell culture, primary monocytes and cell treatment” and “Cell-based models of MAFLD and lipotoxic stress”. Cells were treated with silymarin, silybin, or vehicle (0.5% DMSO or 0.25% ethanol) at 37°C and 5% CO_2_. The pan-kinase inhibitor staurosporine (1 µM) was used as reference compound. After 24 h, 3-(4,5-dimethylthiazol-2-yl)-2,5-diphenyltetrazolium bromide (MTT, 20 µl, 5 mg/ml, Merck) was added to each well, and cells were incubated for another 3 h (HepG2) or 2.5 h (HepaRG) at 37°C and 5% CO_2_ before being lysed in SDS buffer (10% in 20 mM HCl, pH 4.5) overnight. The absorption of the solubilized formazan product was measured at 570 nm (Multiskan Spectrum, Thermo Fisher Scientific or SpectraMax iD3, Molecular Devices).

### Extraction and analysis of phospholipids, neutral lipids, and fatty acids

To extract lipids from cell pellets (HepG2 cells, HepaRG cells, and monocytes) or supernatants of liver homogenates after centrifugation (9,000×g, 10 min, 4°C), PBS pH 7.4, methanol, chloroform, and saline (final ratio: 14:34:35:17) were added in succession 69,70. Phospholipids, TGs and fatty acids in the lower organic phase were evaporated to dryness, dissolved in methanol, and analyzed by UPLC-MS/MS. Internal standards: 1-Pentadecanoyl-2-oleoyl(d7)-sn-glycero-3-phosphoethanolamine, 1-pentadecanoyl-2-oleoyl(d7)-sn-glycero-3-phosphocholine, and/or 1,3-dipentadecanoyl-2-oleyol(d7)-glycerol were used for lipidomic analysis related to Figure 4, Figure 7 and Figure S13 and S14. Other samples contained 1,2-dimyristoyl-*sn*-glycero-3-phosphatidylcholine as internal standard, and 1,2-dimyristoyl-*sn*-glycero-3-phosphatidylethanolamine, 1,2-di-heptadecanoyl-*sn*-glycero-3-phosphatidylglycerol, and/or 1,2-diheptadecanoyl-*sn*-glycero-3-phosphoserine.

Phospholipids, CE, TGs, and free fatty acids were separated on an Acquity^TM^ UPLC BEH C8 column (1.7 μm, 2.1×100 mm, Waters, Milford, MA, USA) using an Acquity^TM^ Ultraperformance LC system (Waters) as described before 71-73. Alternatively, phospholipids and TGs were separated by an ExionLC™ AD UHPLC (Sciex, Framingham, MA, USA) 74-76. In brief, phospholipids were analyzed at a flow rate of 0.75 ml/min at 45°C using acetonitrile/water (95/5) and 2 mM ammonium acetate as mobile phase A and water/acetonitrile (90/10) and 2 mM ammonium acetate as mobile phase B. Mobile phase A was ramped from 75 to 85% within 5 min, followed by an increase to 100% within 2 min and isocratic elution for another 2 min. For the separation of TGs, mobile phase B was replaced by isopropanol, and the initial composition of mobile phase A was lowered from 90 to 70% within 6 min, which was succeeded by isocratic elution for 4 min.

Glycerophospholipids were detected by multiple reaction monitoring (MRM) in the negative ion mode based on their fatty acid anion fragments using a QTRAP 5500 72 or QTRAP 6500^+^ 77. Mass Spectrometer (Sciex), which were equipped with electrospray ionization (ESI) sources. For the analysis of PE and PC using the QTRAP 6500^+^ Mass Spectrometer (Figure 5, Figure S3, and Figure S14), the curtain gas was set to 40 psi, the collision gas was set to medium, the ion spray voltage was set to -4500 V, the heated capillary temperature was set to 650°C (PE) or to 350 °C (PC), the sheath gas pressure was set to 55 psi, the auxiliary gas pressure was set to 75 psi, the declustering potential was set to -50 V, the entrance potential was set to -10 V, the collision energy was set to -38 eV, and the collision cell exit potential was set to -12 V 76.

CE and TGs were identified and quantified in the positive ion mode as NH_4_^+^ adduct ions that undergo neutral loss of either of the acyl groups 73. When using the QTRAP 6500^+^ Mass spectrometer (Figure 4, Figure 5, Figure S3, and Figure S13 and S14), the curtain gas was set to 30 psi (CE) or 40 psi (TG), the collision gas to low, the ion spray voltage to 5500 V, the heated capillary temperature to 350°C (CE) or 400°C (TG), the sheath gas pressure to 55 psi (CE) or 60 psi (TG), the auxiliary gas pressure to 70 psi, the declustering potential to 55 V (CE) 120 V (TG), the entrance potential to 10 V, the collision energy to 22 V (CE) or 35 eV (TG), and the collision cell exit potential to 22 V (CE) or 26 V (TG) 76. Free fatty acids were analyzed by single ion monitoring in the negative ion mode 69 and SM by MRM in the positive ion mode based on the detection of the choline headgroup (m/z = 184)^69^.

Absolute lipid quantities were normalized for Figure 4, Figure 5, S13 and S14 to lipid subclass-specific internal standards and cell number. For other experiments, lipid intensities were normalized to 1,2-dimyristoyl-sn-glycero-3-phosphatidylcholine and the number of cells to calculate the amounts in nmol / 10^6^ cells (PC) or in relative units (other lipid subclasses). Relative intensities represent the percentage of individual lipid species relative to all lipid signals determined within the respective lipid class (= 100%). The most intensive or specific transition was used for quantitation. Analyst 1.6 or Analyst 1.7 (Sciex) were used to acquire and process mass spectra.

### Extraction and analysis of acyl-CoAs

HepG2 cells were suspended in methanol/water (70/30) and placed at -20°C for 1 h. After vigorous mixing, the methanol/water ratio was adjusted to 50/50 and the samples were incubated for another hour at -20°C. Protein precipitates were removed by centrifugation (20,000×g, 5 min, 4°C), and the supernatant was evaporated to dryness. The residue was extracted with methanol/water (50/50) and the extract subjected to UPLC-MS/MS analysis. [^13^C_3_]-Malonyl-CoA (1 nmol; Merck) was used as internal standard.

Acyl-CoAs were separated on an Acquity^TM^ UPLC BEH C18 column (1.7 µM, 2.1×50 mm) with an Acquity^TM^ Ultra Performance LC system 78 and analyzed by MRM in the positive ion mode following electrospray ionization (QTRAP 5500 mass spectrometer). Fragments formed by neutral loss of 2'-phospho-ADP ([M+H-507]^+^) were detected for quantitation. The ion spray voltage was set to 3,000 V, the heated capillary temperature to 600°C, the curtain gas pressure to 30 psi, the sheath gas pressure to 45 psi, the auxiliary gas pressure to 55 psi, the declustering potential to 60 V, the entrance potential to 10 V, and the collision energy to 45 eV (malonyl-CoA) or 30 eV (other acyl-CoAs). Absolute lipid amounts are calculated with respect to the internal standard of the subclass and are normalized to cell number, protein content or tissue weight. Relative lipid proportions are expressed as a percentage of the total sum of all species detected within the corresponding subclass (equal to 100%). Mass spectra were acquired and analyzed using Analyst 1.6 or 1.7 (Sciex).

### Metabolic flux studies

HepG2 cells (1×10^5^ cells/cm^2^) were seeded and directly treated with either vehicle control (0.05% ethanol or 0.1% DMSO), silymarin (10 µg/ml) or silybin A (20 µM) and cultured for 6 h at 37°C and 5% CO_2_. Cells were treated with sodium acetate-^13^C_2_, d_3_ (30 µM, Merck, #299111) for further 18 h before lipids were extracted and analyzed by UPLC-MS/MS as described above. PE species carrying 16:0-^13^C_2_, d_1_, 18:0-^13^C_2_, d_1_, 18:1-^13^C_2_, d_1_, or 20:4-^13^C_2_, d_1_ were quantified by MRM in the negative ion mode as transitions from [M+3+CH_3_COO]^-^ parental ions to the respective isotope-labeled and non-isotope-labeled fatty acid anions. TG species carrying 16:0-^13^C_2_, d_1_, 18:0-^13^C_2_, d_1_, 18:1-^13^C_2_, d_1_, or 18:2-^13^C_2_, d_1_ were detected by MRM in the positive ion mode as transitions from [M+3+NH_4_]^+^ parental ions to the respective fragment anions following release of an isotope-labeled or non-isotope-labeled acyl group. In parallel, non-labeled TG and PE were analyzed to calculate the M+3 isotopic patterns from the monoisotopic signals using the Mass (m/z) calculation tool from Lipid Maps^®^ (https://www.lipidmaps.org/tools/structuredrawing/masscalc.php). These isotopic signals were subtracted from the corresponding signals of the ^13^C_2_, d_1_ - labeled species.

### Transcriptome analysis

Caco-2 cells (1.7×10^5^ cells/cm^2^) were treated with vehicle (0.5% DMSO), 30 µg/ml silymarin or 30 µM silybin for 24 h (n = 3 biological replicates). Total RNA was isolated using a RNeasy Mini Kit (Qiagen) and potential DNA contamination was digested with DNase I (Qiagen) during RNA purification according to the manufacturer's protocol. RNA concentration and quality were assessed using a SpectraMax iD3 microplate reader (Molecular Devices), a bioanalyzer (Agilent) and Qubit (Thermo Fisher Scientific) before being submitted to the MultiOmics Core Facility, Medical University of Innsbruck, for sequencing. The RNA integrity (RIN) of all samples was > 9.5 (out of 10) and no genomic DNA contamination was detected in any of the samples prior to RNA sequencing. Libraries were prepared using Lexogen's Quant Seq 3'mRNA Seq Library Kit FWD with UMI protocol (Lexogen GmbH, Vienna, Austria). Quality validated libraries were multiplexed and sequenced at 150 bp read length using Illumina NovaSeq technology and the generated paired-end raw sequence data reads were quality controlled using FastQC and MultiQC202 79.

Sequencing adapters and reads shorter than 50 base pairs were removed using Trim Galore (Galaxy version 0.6.7) to improve mapping quality, and reads were mapped to the GRCh38 human reference genome (December 2013) using the RNAStar aligner (Galaxy version 2.7.10b) 80. Final transcript count data were generated with HTSeq framework (Galaxy version 2.0.5) 81 for high-throughput sequencing data based on the Ensemble release Homo_sapiens.GRCh38.107 gene annotation with default settings. All analyses were performed on a public instance of Galaxy at usegalaxy.eu. Differential gene expression analysis was performed using DESeq2 package version 1.26 82 with an adjusted *P*-value < 0.05 (5% FDR).

In addition, we re-analyzed microarray-based transcriptome datasets: i) HepG2 cells treated with vehicle (0.0125% DMSO) or 12 µg/ml silymarin (Merck) for 24 h (n = 3 biological replicates)^83^; ii) Huh7.5.1 cells treated with vehicle (0.32% DMSO) or 40 µg/ml silymarin (Madaus Group, Cologne, Germany) for 4, 8, or 24 h (pooled triplicates in three [silymarin, 8 h; silymarin, 24 h], four [vehicle and silymarin, 4 h], or five [vehicle, 8 and 24 h] technical replicates)^84^; iii) primary human hepatocytes from chronically HCV-infected chimeric mice with humanized livers either untreated or receiving 469 mg/kg silybin-C-2',3-bis(hydrogen succinate) disodium salt (Legalon^®^ SIL, in saline, all three mice on day 3 and two mice on day 14) or 265 mg/kg Legalon^®^ SIL (in saline, one mouse on day 14) intravenously daily for 3 or 14 days (n = 3 mice/group) 85. Data are accessible at NCBI GEO database 86, accessions GSE67504, GSE50994, and GSE79103. Differentially regulated genes were identified by pairwise comparison of treatment and control groups using the GEO2R interactive web tool (https://www.ncbi.nlm.nih.gov/geo/geo2r/) 86. *P* values were calculated by multiple *t*-tests, either with or without correction for multiple comparisons according to Benjamini and Hochberg (false discovery rate 5%) and auto-detection for log-transformation.

### Sample preparation, SDS-PAGE, and Western blotting

Pelleted and washed monocytes and HepG2 cells were lysed in ice-cold 20 mM Tris-HCl (pH 7.4), 150 mM NaCl, 2 mM EDTA, 1% Triton X-100, 5 mM sodium fluoride, 10 μg/ml leupeptin, 60 μg/ml soybean trypsin inhibitor, 1 mM sodium vanadate, 2.5 mM sodium pyrophosphate, and 1 mM phenylmethanesulphonyl fluoride, and sonicated on ice (2 × 5 s, Q125 Sonicator, QSonica, Newtown, CT, 125 W, 35% amplitude). After centrifugation (cell lysates: 12,000×g, 5 min, 4°C; liver homogenates: 9,000×g, 10 min, 4°C), the protein concentration of the supernatants was determined using a DC protein assay kit (Bio-Rad Laboratories, CA). Samples (10-15 µg total protein) were combined with loading buffer (1×; 125 mM Tris-HCl pH 6.5, 25% sucrose, 5% SDS, 0.25% bromophenol blue, and 5% β-mercaptoethanol) and heated for 5 min at 95 °C. Proteins were separated by 8-10% SDS-PAGE and transferred to a Hybond ECL nitrocellulose membrane (GE Healthcare) or Amersham Protran 0.45 µm NC nitrocellulose membranes (Carl Roth, Karlsruhe, Germany). Membranes were blocked with 5% bovine serum albumin (BSA) or skim milk for 1 h at room temperature and incubated with primary antibodies overnight at 4°C. IRDye 800CW-labeled anti-rabbit IgG (1:10,000, 92632211, LI-COR Biosciences, Lincoln, NE), IRDye 800CW-labeled anti-mouse IgG (1:10,000, 926-32210, LI-COR Biosciences, Lincoln, NE), IRDye 680LT-labeled anti-rabbit IgG (1:80,000, 926-68021, LI-COR Biosciences, Lincoln, NE), IRDye 680LT-labeled anti-mouse IgG (1:80,000, 926-68020, LI-COR Biosciences, Lincoln, NE), DyLight^®^ 680 goat anti-rabbit IgG (1:10,000, # 35569, Thermo Fisher Scientific), and/or DyLight^®^ 800 goat anti-mouse IgG (1:10,000, # SA5-10176, Thermo Fisher Scientific) were used as secondary antibodies. Fluorescent, immunoreactive bands were visualized using an Odyssey infrared imager (LI-COR) or a Fusion FX7 Edge Imaging System (spectra light capsules: C680, C780; emission filters: F-750, F-850; VILBER Lourmat, Collegien, France) 74. Acquired data from densitometric analysis were linearly adjusted and background-corrected using Odyssey Infrared Imaging System Application Software Version 3.0 (LI-COR Biosciences) or Evolution-Capt Edge Software Version 18.06 (VILBER Lourmat) and Bio-1D imaging software Version 15.08c (Vilber Lourmat), and protein levels were normalized to GAPDH or β-actin.

### qPCR

HepG2 cells were incubated with silymarin (10 µg/ml), silybin (20 µM), or vehicle (ethanol for silymarin, DMSO for silybin) for 24 h. Total RNA of HepG2 cells was isolated with the E.Z.N.A Total RNA Kit (Omega Bio-tek, Norcross, GA). SuperScript III First-Strand Synthesis SuperMix (Thermo Fisher Scientific) was used for transcription into cDNA. The cDNA was snap-frozen and stored at -20 °C until use. An aliquot of the cDNA preparation (1.25 µl) was combined with 1× Maxima SYBR Green/ROX qPCR Master Mix (Fermentas, Darmstadt, Germany) and forward and reverse primer (0.5 µM; TIB MOLBIOL, Berlin, Germany) in Mx3000P 96-well plates. Primer sequences are given in Table 1. β-Actin and GAPDH were used as reference. PCR was performed on a StraTGene Mx 3005P qPCR system (Agilent Technologies, Santa Clara, CA). The PCR program heats to 95°C for 10 min and conducts 45 cycles of 15 s at 95°C, 30 s at 61°C, and 30 s at 72°C. Threshold cycle values were determined by MxPro Software (Mx3005P/version 4.10, Agilent Technologies) and normalized to the amount of total RNA.

### Immunofluorescence microscopy

HepG2 cells (2.5×10^4^/3.9 cm^2^) were seeded on an ibidi 8-well slide (ibidi #80826, Gräfelfing, Austria) and cultured for 24 h at 37 °C and 5% CO_2_. For ER and lipid droplet staining, vehicle (0.1% DMSO for silybin A or 0.05% ethanol for silymarin), silymarin, or silybin were added, and cells were incubated for another 24 h at 37 °C and 5% CO_2_. The medium was then removed, and the cells were rinsed twice with HBSS. Prewarmed BioTracker™ 488 Green Lipid Dye Biotracker (Merck, # SCT144, 1x in HBBS /Ca/Mg, Gibco cat. #14025-092) or ER-Tracker™ Red (BODIPY™ TR Glibenclamide) (ThermoFisher Scientific, Vienna, Austria #E34250, 1 µM in HBSS) staining solutions were added and cells incubated for 30-60 min before being washed with HBSS. Hoechst DNA staining solution was applied (Merck, # 33258, 1 μg/ml) and cells incubated for 30 min at 37 °C and 5% CO_2_. The staining solution was then removed and the cells were fixed with 4% paraformaldehyde in H_2_O for 20 min, followed by two washes with HBSS. Fresh HBSS buffer was added and the cells were immediately visualized by fluorescence microscopy. For Golgi staining, cells were incubated with vehicle (0.1% DMSO for silybin A or 0.05% ethanol for silymarin), silymarin or silybin for 6 h at 37 °C and 5% CO_2_. The cells were rinsed twice with HBSS and 2 μL of the BacMam 2.0 reagent CellLight™ Golgi-GFP (ThermoFisher Scientific, Vienna, Austria, # C10591) was added, followed by incubation at 37°C in 5% CO_2_ for 18 h. Cells were washed with HBSS twice, Hoechst DNA stain solution was added and cells were incubated for 30 min at 37 °C and 5% CO_2_. Fluorescently labelled organelles were visualised using a BZ-X800E fluorescence microscope (Keyence, Neu-Isenburg, Germany) equipped with the BZ-X Filters DAPI (OP-87762, λ_ex_ = 360 nm, λ_em_ = 460 nm), GFP (OP-87763, λ_ex_ = 470 nm, λ_em_ = 525 nm) and TRITC (OP-87764, λ_ex_ = 545 nm, λ_em_ = 605 nm) and a Plan Apochromat 40× (NA 0.95) objective. Images were captured using the sectioning module with structured illumination and z-stacks of 10 µM. Image analysis was performed using ImageJ software (https://imagej.net/ij/). For quantification of ER and Golgi, a region of interest (ROI) was drawn around the labeled organelles, and the mean intensity was measured. Only cells within the focal plane were considered, defined as cells in which the ER appeared as a perinuclear ring surrounding at least 50% of the nucleus or in which Golgi signals appeared as distinct, well-defined spots. Lipid droplet quantification was performed by setting a minimum threshold of 15 to exclude background staining. Number and size of lipid droplets were automatically measured using the "Analyze Particles" tool in ImageJ.

### Immunohistochemistry (IHC)

Liver samples were immediately fixed in neutral buffered 4% paraformaldehyde for at least 24 h and then dehydrated in increasing alcohol concentrations, embedded in paraffin, and sliced into 4 µm sections as described before 87. The sections were deparaffinized with xylene and rehydrated using an inverse series of aqueous alcohol concentrations. Hydrogen peroxide (0.3% in methanol) was applied for 45 min to block endogenous peroxidase activity.

Sections were microwaved in citric acid (10 mM, pH 6.0) for 16 min at 600 W and then incubated with primary antibodies (mouse anti-GRP78, 1:5000; goat anti-rat CYP1A1, 1:5000; goat anti-rat CYP3A2, 1:5000; goat anti-rat CYP2B1, 1:5000) in PBS pH 7.4 and 5% BSA overnight at 4°C, followed by treatment with secondary biotinylated rabbit anti-goat IgG or rabbit anti-mouse IgG (30 min, room temperature) and peroxidase-conjugated avidin (VECTASTAIN^®^ Elite ABC-Kit; Vector Laboratories, Burlingame, CA; another 30 min). The chromogen 3-amino-9-ethylcarbazole (AEC Substrate Pack; BioGenex, San Ramon, CA) was applied twice for 15 min to visualize immunoreactive sites. Sections were mounted in Vectamount™ mounting medium (Vector Laboratories, Burlingame, CA) and analyzed using an Axio Imager A1 microscope equipped with a 20× objective and a ProgRes C5 camera (Jenoptik, Jena, Germany).

### Animal housing and treatment of mice with silybin hemisuccinate

Male C57BL/6 mice (12-weeks-old, body weight 25-30 g; Charles River, Sulzfeld, Germany) were housed under standardized conditions with a day-night cycle of 12 h/12 h at 22 ± 1°C and 50 ± 10% environmental humidity. Standard diet and water were provided *ad libitum*. Animals were adapted to laboratory conditions before the experiment for at least 2 days. Silybin hemisuccinate (200 mg/kg) or vehicle (0.9% NaCl) were intraperitoneally administered trice (at 0, 12, and 24 h). Mice were anesthetized by isoflurane and sacrificed by isoflurane overdose after 37 h, and organs were removed, weighed and either fixed in 10% buffered formaldehyde or snap-frozen in liquid nitrogen for biochemical analysis. All experiments were performed in accordance with the German legislation on protection of animals and with approval of the Thuringian Animal Protection Committee.

### GSH and GSSG levels

The tissue content of glutathione in its reduced (GSH) and oxidized (GSSG) form was analyzed by homogenizing the liver and kidney samples with eleven volumes of 0.2 M sodium phosphate buffer (5 mM EDTA; pH 8.0) and four volumes of 25% metaphosphoric acid. After centrifugation (12,000×g, 4°C, 30 min), the GSH content was measured in the supernatants using a colorimetric assay as previously described 88. The GSSG concentration was assessed fluorometrically 89.

### Lipid peroxidation

To determine the tissue content of lipid peroxides as thiobarbituric acid reactive substances (TBARS), liver and kidney samples were homogenized in 19 volumes of ice-cold saline and analyzed fluorometrically 90.

### Biotransformation capacity

To obtain 9,000×g supernatants, the livers were homogenized in 0.1 M sodium phosphate buffer (pH 7.4) (1:2 w/v) and subsequently centrifuged at 9,000×g for 20 minutes at 4°C. Activities of all biotransformation reactions were assessed in these 9,000×g supernatants and referred to the protein content of this fraction which was determined with a modified Biuret method 91. For assessment of CYP enzyme activities, the following model reactions were performed: benzyloxyresurofin-*O*-debenzylation (BROD) 92, ethoxycoumarin-*O*-deethylation (ECOD) 93, ethoxyresorufin-*O*-deethylation (EROD) 94, ethylmorphine-*N*-demethylation (EMND) 95, methoxyresorufin-*O*-demethylation (MROD) 94, pentoxyresorufin-*O*-depentylation (PROD) 94. GST activities were determined using *o*-dinitrobenzene as a substrate. The resulting dinitrobenzene-glutathione conjugate was measured photometrically 96. For the determination of UGT activities, 4-methylumbelliferone was used as a substrate and the respective glucuronide was measured fluorometrically 97,98.

### Blood glucose levels

Blood glucose levels were determined using a commercially available blood glucose meter and respective test strips (BG star1, Sanofi-Aventis, Frankfurt, Germany).

### Data analysis and statistics

Data are given as individual values and/or means ± SEM or + SEM of *n* independent experiments. Statistical analysis was performed with GraphPad Prism 8.3 or 9.0 (GraphPad Software Inc, San Diego, CA, USA) using non-transformed or logarithmized data. Ordinary or repeated-measures one-way ANOVAs followed by Tukey *post-hoc* tests were applied for multiple comparison, and two-tailed Student's *t*-tests were used for paired and unpaired observations (two-sided α levels of 0.05). Statistical significance was defined as *P < 0.05, **P < 0.01, and ***P < 0.001. Outliers were determined by Grubb's test. Figures were created with Graphpad Prism 8.3 or 9.0 (GraphPad Software Inc), Excel 2016 or 2020 (Microsoft, Redmond, WA), or Sigma Plot 13.0 (Systate Software GmbH, San Jose, CA).

## Results

### Silybin induces a switch from hepatic TGs to phospholipids

To investigate the effects of silymarin and silybin on the hepatic lipid composition, we monitored concentration- and time-dependent changes in PE levels in HepG2 cells by targeted lipidomics. Phospholipid accumulation in HepG2 cells was manifested at ≥ 10 µg/ml silymarin or 20 µM silybin after 24 h (Figure S1), and cytotoxic activities first became evident at ≥ 50-200 µg/ml silymarin and ≥ 100 µM silybin (Figure S2). For the following experiments, human HepG2 hepatocarcinoma cells and human primary monocytes (as a surrogate for hepatic phagocytes) were used and treated with 50 µg/ml silymarin for monocytes, 10 µg/ml silymarin for HepG2 cells and 20 µM silybin for 24 h. Silymarin increased the cellular content of major phospholipid classes, i.e., PC, PE, phosphatidylserine (PS), phosphatidylinositol (PI), phosphatidylglycerol (PG), and SM (Figure 1B and C). Similar effects were observed for silybin (20 µM, Figure 1B and C), one of the major bioactive components of silymarin 43. Instead, TG levels were substantially decreased by both silymarin and silybin treatment, with opposite efficacy in monocytes and hepatocytes. While silymarin specifically reduced TG levels in monocytes, silybin was only effective in hepatocytes (Figure 1B and C). Together, our results suggest that silybin induces a hepatic switch from TGs to phospholipids and point to additional components contained in silymarin that tune the cellular lipid profile.

To investigate whether the decrease in TGs is functionally related to the accumulation of phospholipids, we studied the impact of TG degradation on the cellular PE content, which was robustly upregulated by silybin treatment (Figure 1C). The selective diacylglycerol-*O*-acyltransferase (DGAT)2 inhibitor PF-06424439 (10 µM), which interferes with the final step of TG biosynthesis 99, decreased TG levels as expected, but failed to increase the amount of PE (Figure S3). Accordingly, inhibition of adipocyte triglyceride lipase (ATGL) using atglistatin neither decreased TG nor significantly elevated PE levels (Figure S3). Thus, our data suggest that the reduction in TGs does not account for the enrichment in phospholipids, at least under conditions where phospholipid biosynthesis is not upregulated.

Next, we investigated whether silybin counter-regulates phospholipid and TG levels *in vivo*. Mice received silybin (200 mg/kg, i.p.) three times over 37 h, which is expected to produce peak hepatic concentrations >10 nmol/g for the unconjugated drug 100,101. Silybin increased the hepatic phospholipid content, reaching significance for PE, PS, and PI, and simultaneously lowered TG levels (Figure 1D), as expected from the results for hepatocytes *in vitro*. The shift from TGs to phospholipids was accompanied by a significant loss of liver and body weight (Figure 1E) and a decrease of blood glucose levels (Figure S4), which is of particular interest because fatty liver disease is often associated with insulin resistance that elevates blood glucose levels 3. Note that the mice were fed ad libitum and food intake was not measured. Therefore, it cannot be excluded that the observed effects of silybin may be partially related to reduced food intake.

**Figure 2:**
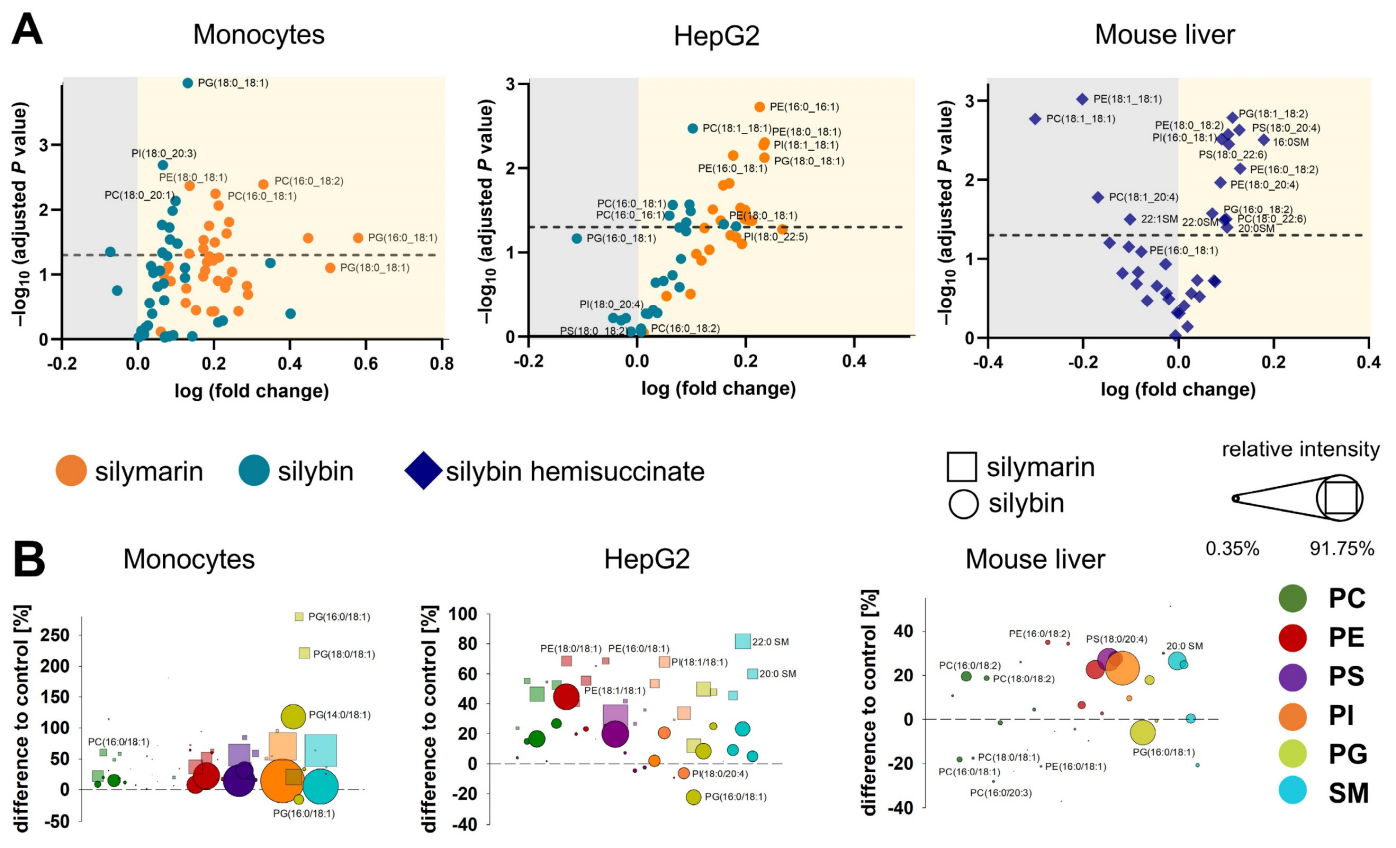
Phospholipid profiling indicates an upregulation of diverse species. Human primary monocytes and HepG2 cells were treated with silymarin (50 µg/ml for monocytes and 10 µg/ml for HepG2 cells), silybin (20 µM) or vehicle (ethanol for silymarin, DMSO for silybin) for 24 h. Mice received silybin hemisuccinate ('silybin'; 200 mg/kg) or vehicle (0.9% NaCl) trice at 0, 12, and 24 h and were sacrificed after 37 h. (A) Volcano plots showing the cellular proportion of phospholipid species that increase (yellow background) or decrease (grey background) upon treatment with silymarin or silybin. Adjusted *P* values given vs. vehicle control. The dashed line indicates a *P*-value of 0.05. (B) Forest plots depicting phospholipid species that are up- (positive values) or down-regulated (negative values) by silymarin (squares) or silybin (circles). Values, calculated as percentage of control, show the difference to 100%, with the dashed line at 0% indicating no difference to control. The dot size describes the mean relative abundance of phospholipid species within the phospholipid subclass (relative intensities). Data and the number of experiments are identical to Figure 1.

The majority of phospholipids significantly upregulated by silybin in mouse liver contain polyunsaturated fatty acids, either linoleic acid (18:2), arachidonic acid (20:4), or docosahexaenoic acid (22:6) (Figure 2A). Note that an increase in membrane unsaturation has been associated with insulin sensitivity 102 and may explain the decrease in blood glucose levels with silybin administration (Figure S4). The effect of silymarin/silybin on individual lipid species varies greatly between experimental systems (Figure S5 and S6). While the levels of a broad spectrum of phospholipid species are increased, there are also lipids that are regulated in the opposite direction, particularly in mouse liver, where silybin reduces the amount of PC (18:1/18:1) and PE (18:1/18:1), along with other lipids (Figure 2A and Figure S6). The differences between silymarin and silybin lie in the magnitude rather than the direction of the phospholipidomic changes (Figure 2B). In contrast, the levels of TG species are consistently decreased by silymarin in monocytes and by silybin in HepG2 cells (Figure S5). To exclude the possibility that lipids present in silymarin contribute to changes in the cellular lipid profile, we analyzed the lipid composition of silymarin. Phospholipids with a glycerol backbone (glycerophospholipids) other than PC (16:0/18:2) were not detected in silymarin, and only low-abundance lysophospholipid and SM species were present (Figure S7). Together, the lipids in silymarin do not explain the increase in cellular phospholipids upon treatment.

### Accumulated phospholipids are distributed across intracellular membranes

Phospholipids are organized in plasma and intracellular membranes and, to a lesser extent, in lipid droplets and the cytosol 13,103. It can be excluded that the silymarin/silybin-induced increase in cellular phospholipids is related to the plasma membrane, as the diameter of both monocytes and HepG2 cells was not altered by treatment (Figure S8A). To define the membrane compartment where the additional phospholipids are deposited, we assessed their size and morphology using organelle-specific fluorescence probes (Figure S8). We expected that the 1.2- to 1.5-fold increase in total intracellular phospholipids would be visible as a gain in size or morphological change if the additional phospholipids were preferentially incorporated into a specific membrane compartment. If, instead, the phospholipids are evenly distributed throughout the intracellular membranes, even the 1.5-fold increase in spherical surface area (formed by membrane phospholipids) would result in only a 1.2-fold increase in diameter, and this factor is further reduced for tubular systems such as ER and Golgi with strongly increased surface areas as compared to spherical structures. Apparent effects on organelle size and structure (as assessed by quantitative analysis of the fluorescence probes) are unlikely to be achieved in this case. We focused on large intracellular membrane compartments, i.e., nucleus, ER, and Golgi, which were stained with Hoechst DNA stain and live cell dyes for ER and Golgi, respectively. Silymarin/silybin A did not markedly affect the intensity or distribution of the fluorescence signal (Figure S8B and C), as confirmed by quantitative analysis of the fluorescence signal (Figure S8B and C). Thus, phospholipids seem to be enriched at intracellular sites but not preferentially incorporated into a major membrane compartment such as the ER, Golgi, or nucleus.

### Silybin causes a decrease in lipid content

Lipid droplets are universal storage organelles for neutral lipids such as TG and cholesteryl esters (CE) and represent dynamic cellular organelles with an important role in lipid and membrane homeostasis 13. We treated HepG2 cells with silymarin or silybin (A) and stained lipid droplets with either Oil Red O or BioTracker™ 488 Green Lipid Dye. Spectroscopic analysis of lipid droplets, quantifying the incorporated Oil Red O (Figure S8E), showed that silybin reduced their content. Interestingly, this reduction was not due to a decrease in the number of lipid droplets, but rather appeared to result from a decrease in their size (based on image quantification of cells stained with the BioTracker Lipid Dye) (Figure S8D). These findings are consistent with the observed decrease in TG levels (Figure 1C) as well as with previous *in vitro* and *in vivo* studies using silymarin or silybin 104-108. Silymarin was considerably less efficient in reducing TG levels (Figure 1C), and lipid droplet content in HepG2 cells (Figure S8D and E).

### Stereochemical requirements of silybin for targeting lipid metabolism

**Figure 3:**
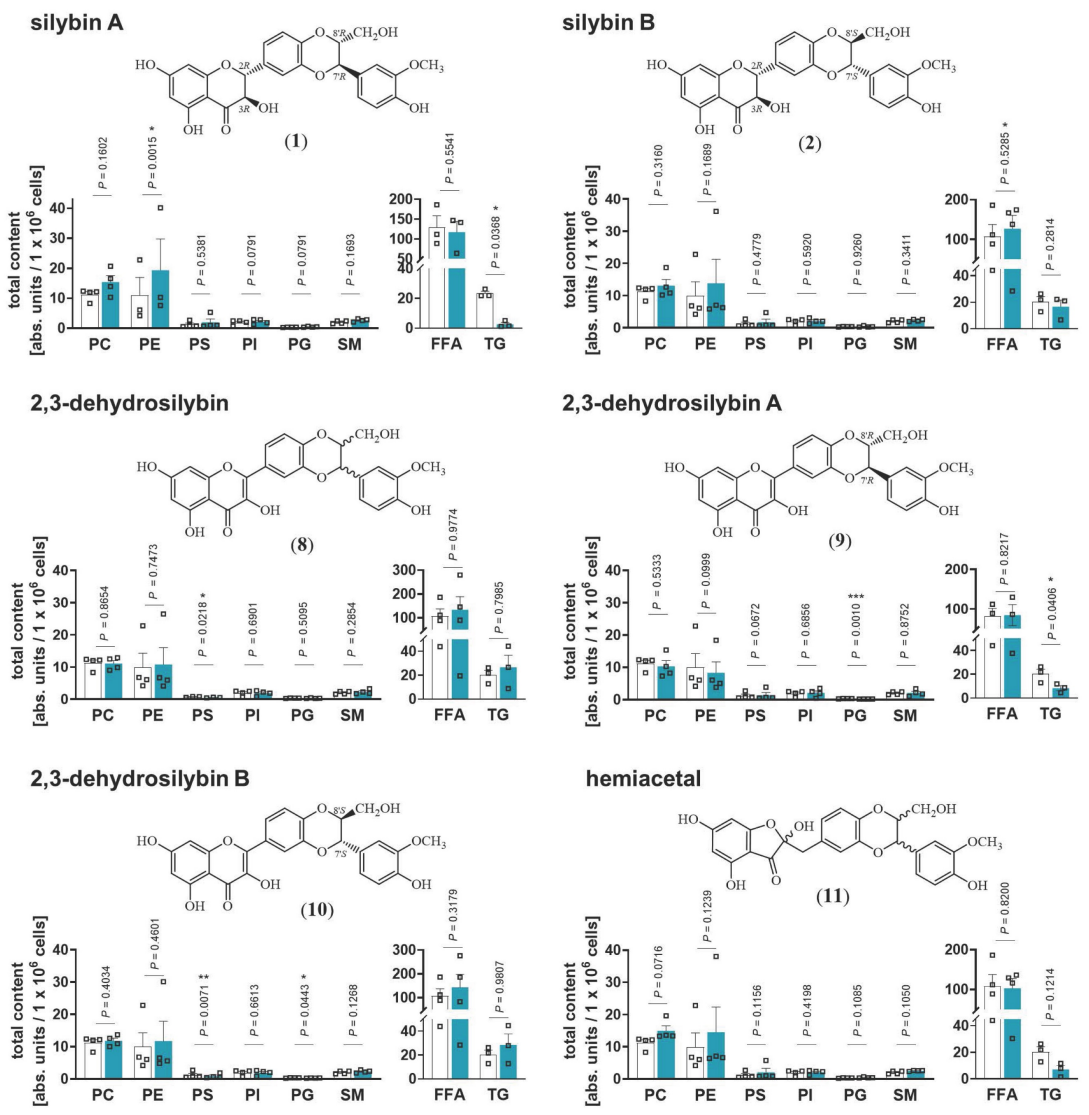
Silybin A is the active isomer that causes the switch from TGs to phospholipids. HepG2 cells were treated with the indicated compounds (20 µM) or vehicle (DMSO) for 24 h. Total amounts of lipid classes were determined by UPLC-MS/MS and are given as nmol / 1×10^6^ cells for PC and units / 1×10^6^ cells for PE, PS, PI, PG, SM and TG. Individual values and mean + SEM; n = 3 (PE silybin A, PS dehydrosilybin, TGs, free fatty acids (FFA) 2,3-dehydrosilybin A and B) n = 4 (except PE silybin A, PS dehydrosilybin, TGs, free fatty acids FFA 2,3-dehydrosilybin A and B). *P < 0.05, **P < 0.01, ***P < 0.001 vs. vehicle control (DMSO). Two-tailed paired Student's *t*-test of log-transformed data.

Natural silybin is a mixture of the diastereoisomers silybin A and B 43. To elucidate the active isomer and explore crucial structural features, we applied an efficient preparative HPLC method to obtain the two isomers A and B in pure form 65. Starting from these isomers, the corresponding 2,3-dehydrosilybin enantiomers and the hemiacetal product, in which the 2,3-dihydro-chromane is replaced by 2*H*-benzofuran-3-one, were synthesized 66 (Figure 3). Lipidomic analysis revealed that silybin A increased phospholipid and decreased TG levels in HepG2 cells, whereas silybin B was considerably less effective (Figure 3). Introduction of a double bond into the flavanon-3-ol moiety of silybin yielded 2,3-dehydrosilybin, which (as *7'R,8'R* isomer A) decreased TG levels comparably to silybin but was no longer active on phospholipids (Figure 3). These findings indicate that both, the 2,3-dihydrochromane and the 1,4-benzodioxan scaffold of silybin A contribute to the phospholipid-accumulating activity, whereas modifications of the 2,3-dihydrochromane ring are compatible with TG-lowering properties. Hence, silybin seems to modulate TG and phospholipid metabolism through independent mechanisms. 2,3-Dehydrosilybin and its isomers A and B (Figure 3) selectively decreased the abundance of anionic phospholipids. On the one hand, 2,3-dehydrosilybin lowered the cellular PS content, which we ascribed to isomer B. On the other hand, both isomers, but surprisingly not the stereomeric mixture, induced a drop of PG (2,3-dehydrosilybin A > 2,3-dehydrosilybin B), the precursor of cardiolipins 64. The hemiacetal (Figure 3) increased phospholipid and decreased TG levels by trend, being slightly less efficient than silybin A (Figure 3**)** but more active than the stereomeric mixture of silybin (Figure 1C). Neither cell number nor membrane integrity were substantially reduced by any of the silybin derivatives up to 20 µM (Figure S9). Together, the effects of silybin on the cellular lipid profile are mediated by only one isomer, and small changes in its structure allow to dissect the activities on phospholipids and TGs.

### Silymarin/silybin acts on multiple nodes in the lipid metabolic network, reducing the overall expression of enzymes involved in triglyceride biosynthesis and phospholipid degradation

To elucidate the molecular mechanisms by which silymarin/silybin induces a lipid class switch from TGs to phospholipids, we reanalyzed previously published transcriptomic datasets from hepatocytes (*in vitro* and *in vivo*) and acquired the transcriptome of an exemplary extrahepatic cell line to distinguish liver-specific from general effects. We focused on genes from the category “Lipid Metabolism” of the Reactome Pathway Database 109 and studied their expression in four experimental systems *in vitro* and *in vivo*: i) human HepG2 hepatocarcinoma cells treated with silymarin (12 µg/ml) for 24 h 83, ii) human Huh7.5.1 hepatocarcinoma cells treated with silymarin (40 µg/ml) for 4, 8, and 24 h 84, iii) human Caco-2 colon carcinoma cells treated with either silybin (30 µM) or silymarin (30 µg/ml) for 24 h, and iv) hepatocytes isolated from chronically hepatitis C virus (HCV)-infected mice receiving daily intravenous injections of silybin (265-469 mg/kg) for 3 or 14 d 85. Silybin/silymarin affects the expression of a wide range of enzymes and factors involved in lipid metabolism, but the effects are moderate and, with one exception, do not reach significance after global correction for false discovery (Figure S10A-D). Only the cytochrome P_450_ (CYP) monooxygenase *CYP1A1*, which accepts various endogenous substrates, including steroids and polyunsaturated fatty acids 110-114, is highly significantly upregulated in Caco-2 cells (Figure S10C).

### Silymarin/silybin induces the expression of enzymes involved in phospholipid biosynthesis, while reducing the expression of phospholipid degradation enzymes

**Figure 4:**
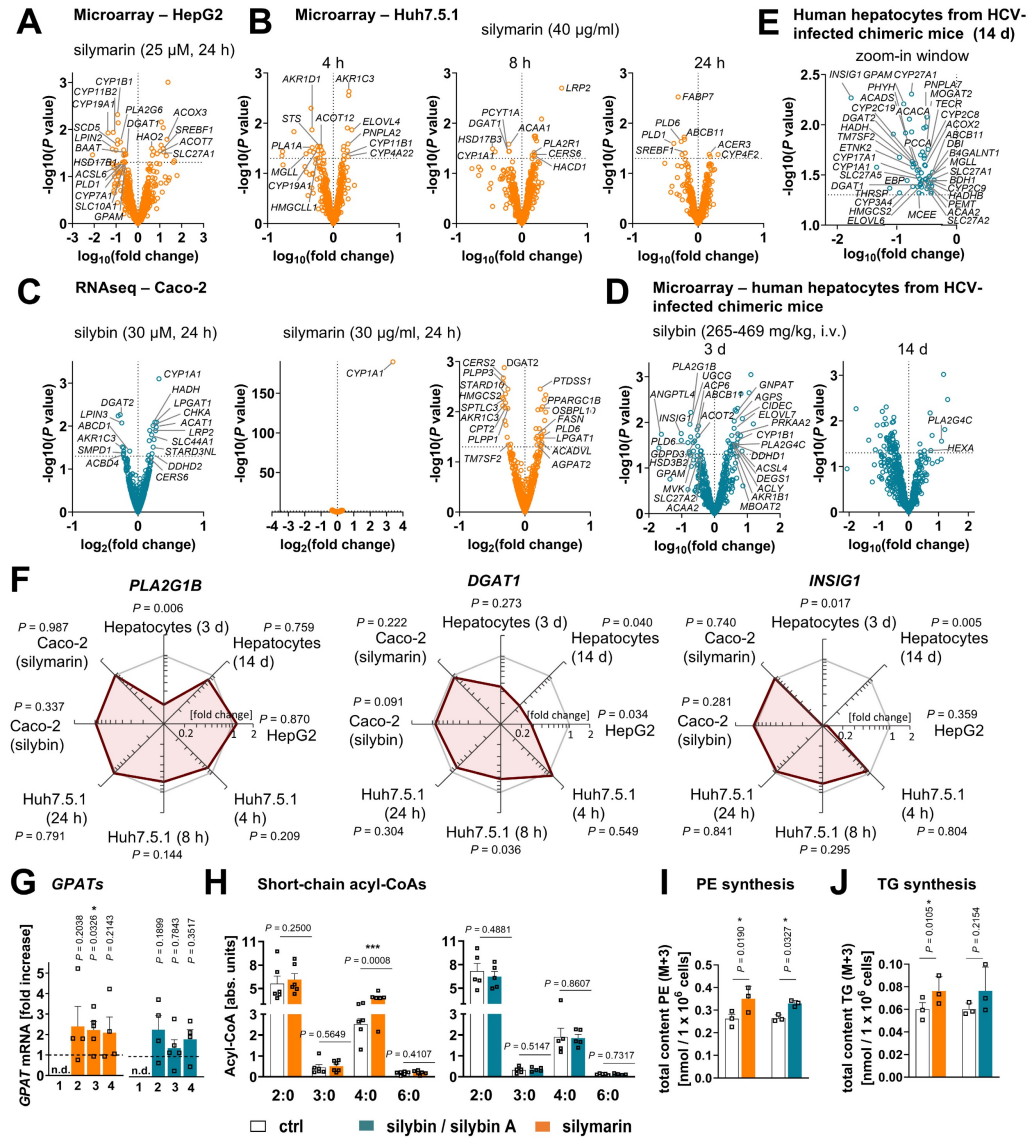
Silymarin/silybin induces global changes in phospholipid, TG and sterol metabolism. (A-F) Comparative analysis of transcriptome data from silymarin-treated HepG2 (A) and Huh7.5.1 hepatocarcinoma cells (B), silymarin- and silybin-treated Caco-2 colon carcinoma cells (C), and hepatocytes derived from HCV-infected mice receiving silybin (D, E). Volcano plots compare the expression of lipid metabolic genes upon silymarin (A-C) or silybin (C-E) treatment vs. vehicle control. Differentially expressed genes are defined as those that show consistent regulation in the same direction in at least two independent model systems at a significance level of *P* < 0.05 (without adjustment for multiple comparisons) and are annotated in the corresponding plots. The dashed line indicates a *P*-value of 0.05; multiple two-tailed unpaired Student's *t*-tests. (F) Radar plots indicating the fold change in *PLA2G1*, *DGAT1*, and *INSIG1* expression by silymarin (HepG2, Huh7.5.1, Caco-2) or silybin (hepatocytes, Caco-2) relative to vehicle control. Non-adjusted *P* values given vs. vehicle control; multiple two-tailed unpaired Student's *t*-tests (G-J). HepG2 cells were incubated with silymarin (10 µg/ml), silybin (20 µM) or vehicle (ethanol for silymarin, DMSO for silybin) for 24 h. (G) mRNA levels of *GPAT2-4* normalized to β-actin. Individual values and mean + SEM as fold-change of control; n = 4 (GPAT2 and GPAT4), n = 5 (*GPAT3*). (H) Effects of silymarin and silybin on the cellular ratio of short-chain acyl-CoAs, normalized to the internal standard [^13^C_3_]-malonyl-CoA. Individual values and mean + SEM; n = 5 (silybin) and n = 6 (silymarin). *P < 0.05, ***P < 0.001 vs. vehicle controls; two-tailed paired Student's *t*-tests. (I, J) Incorporation of isotopically labeled sodium acetate-^13^C_2_, d_3_ in PE (I) and TG (J) by HepG2 cells treated with silymarin (10 µg/ml), silybin (20 µM), or vehicle (ethanol for silymarin, DMSO for silybin) for 24 h. The total amount of the isotopically labeled PE and TG species analyzed is shown. Individual values and means + SEM; n = 3. *P < 0.05 vs. vehicle controls; two-tailed paired Student's *t*-tests.

Given the detected changes in the HepG2 lipidome (Figure 1B-D), we extended our study to genes that were differentially regulated according to non-adjusted *P*-values and for which the respective pathway was significantly regulated in the same direction for at least two independent model systems. We found that silybin/silymarin i) decreased the expression of several lipases involved in phospholipid degradation (Figure 4A-F), including phospholipases A_1_ (*PLA1A*, Figure 4B), phospholipases A_2_ (*PLA2G1B, PLA2G6*, Figure 4A and D, and Figure S11), and phospholipase D (*PLD1, PLD6*, Figure 4A, B, and D, and Figure S11), specifically in primary hepatocytes and hepatocyte-derived cell lines.

In addition, silybin/silymarin ii) upregulates factors that deplete phospholipases (*PLA2R1*, Figure 4B), iii) downregulates enzymes that degrade intermediates in phospholipid biosynthesis (*TECR, MGLL, ACP6, GDPD3, PNPLA7*, Figure 4B, D and E), and iv) less consistently induces the expression of phospholipid biosynthetic enzymes and other factors (*GNPAT, CHKA, SLC44A1, AGPS, AGPAT2, MBOAT2, LPGAT1, DEGS1, CERS6*, Figure 4C and D and Figure S11).

Compensatory mechanisms seem to exist that decrease phospholipid biosynthesis (*via PCYT1A, ETNK2, PEMT, GPAM, SPTLC3, CERS2*, Figure 4B, C, D and E) or enhance phospholipid degradation (*PLA2G4C, DDHD1, ACER3, PLD6*, Figure 4B, C and D), possibly buffering the accumulation of phospholipids or rearranging phospholipid profiles through different substrate specificities.

To investigate whether silymarin/silybin elevates phospholipid levels *via de novo* phospholipid biosynthesis under our experimental conditions, we treated HepG2 cells with silymarin or silybin for 24 h and determined the mRNA expression of glycerophosphate acyltransferase (*GPAT*) isoenzymes and lysophosphatidic acid acyltransferase (*LPAAT*)/lysophospholipid acyltransferase (*LPLAT*) isoenzymes at the mRNA level. *GPATs* and *LPAATs* successively transfer acyl-chains from acyl-CoA to the *sn*-1 and *sn*-2 positions of glycerol-3-phosphate to form phosphatidic acid, the common precursor of glycerophospholipids and TGs 64. Silymarin and silybin increased the mRNA levels of *GPAT* isoenzymes 2 to 4, reaching significance for the silymarin-mediated induction of *GPAT3* (Figure 4G), which is consistent with a previous report showing enhanced *Gpat3* mRNA expression in the liver of silybin-treated mice on a methionine- and choline-deficient diet 104. In contrast, the expression of *LPAAT*/*LPLAT* isoenzymes was not markedly affected (Figure S12A). Together, the moderate but versatile induction of phospholipid biosynthesis and inhibition of phospholipid degradation by silymarin/silybin likely accounts for the accumulation of phospholipids in hepatocytes.

### Silymarin/silybin reduces the expression of triglyceride-synthesizing enzymes

**Figure 5:**
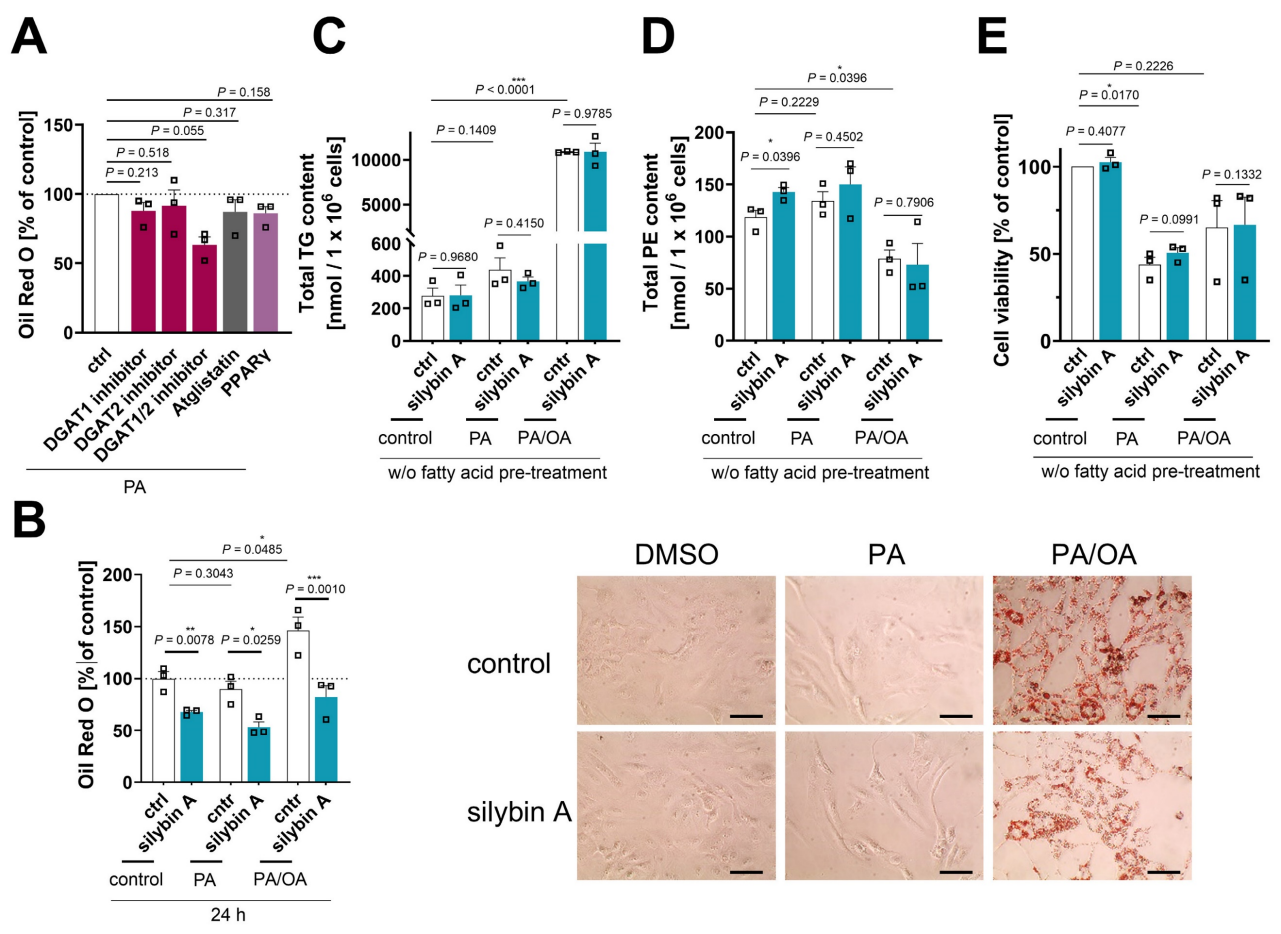
The efficacy of silybin in inducing a lipid class switch differs between hepatocyte pre-disease and disease models. (A,B) HepaRG cells were treated with 0.1 mM palmitate (PA) or a mixture of PA/oleate (OA) in a 1:2 ratio (in total 1 mM) together with vehicle (DMSO, 0.5%), silybin A (20 μM), the ATGL inhibitor atglistatin (50 µM), the DGAT1 inhibitor A 922500 (5 µM), the DGAT2 inhibitor PF-06424439 (10 µM), a combination of DGAT1 (5 µM) and DGAT2 inhibitors (10 µM), or the PPARγ antagonist GW9662 (5 µM) for 24 h. (A) Relative lipid droplet content. Individual values and mean + SEM, n = 3. (B) Left panel: Relative lipid droplet content. Individual values and mean + SEM, n = 3. Right panel: Representative images of HepaRG cells stained for lipid droplets using Oil Red O; scale bar, 50 µm. (C, D) HepaRG cells were co-treated directly with with 0.1 mM palmitate (PA) or a mixture of PA/oleate (OA) in a 1:2 ratio (in total 1 mM) and vehicle (DMSO, 0.5%) or silybin A (20 μM) for 24 h. Total levels of TG (C) and PE (D) determined by UPLC-MS/MS. Individual values and mean + SEM, n = 3. (E) Cell viability measured by MTT assay. Individual values and mean + SEM, n = 3. *P < 0.05, **P < 0.01, ***P < 0.001 vs. control; two-tailed paired (A, B, E) or unpaired (C, D) Student's *t*-test.

The decrease in TG levels is driven by the repression of genes associated with the generation of DAGs from either phosphatidate (*LPIN2, LPIN3, PLPP1, PLPP3*, Figure 4A and C, and Figure S11) or monoacylglycerols (*MOGAT2*, Figure 4E) and their acylation to TGs (*DGAT1, DGAT2*, Figure 4A, B, C and E, and Figure S11), as suggested by comparative transcriptomics. The concrete mode of action seems to be context-dependent and possibly under kinetic control, as suggested by the failure of silybin and silymarin to reduce DGAT1 and DGAT2 protein expression in HepG2 cells 24 h after treatment (Figure S12B). TGs are a major component of the hydrophobic core of lipid droplets, which form contact sites with essentially all other cellular organelles and are at the nexus of lipid and energy metabolism 13,115,116. Interestingly, selective inhibition of DGAT1 (by A-922500) or DGAT2 (by PF-06424439) and antagonism of the DGAT-inducing transcription factor peroxisome proliferator activated receptor (PPARγ) 117 (by GW9662) moderately reduced lipid droplet staining in palmitate (PA, 16:0)-loaded human HepaRG hepatocytes (Figure 5A), but only the combined inhibition of DGAT1 and DGAT2 reached the efficacy of the silybin isomer A (Figure 5B). Since lipolysis of TGs in lipid droplets is initiated by ATGL/PNPLA2 118, we investigated the effect of silymarin/silybin on the protein expression of this enzyme, but again found no substantial regulation (Figure S12B), consistent with the transcriptomics data (Figure 4A-E). Note that selective inhibition of ATGL (by atglistatin) also failed to increase lipid droplet signals in stressed HepaRG cells (Figure 5A).

### Silymarin/silybin causes subtle changes in fatty acid anabolism

Both phospholipid and TG biosynthesis depend on the availability of activated fatty acids 119. Their biosynthesis from acetyl-CoA is an energy- and NADPH-consuming process, which is initiated by the rate-limiting enzyme acetyl-CoA carboxylase (ACC, *ACACA*) 120. The product of this reaction, malonyl-CoA, is subsequently transferred to fatty acid synthase (*FASN*), which produces long-chain fatty acids that are activated as CoA esters by acyl-CoA synthetases before further metabolism 121,122. As expected from the multiple roles of acyl-CoAs in lipogenesis, silymarin/silybin ambiguously regulates genes related to fatty acid metabolism, with expression changes either promoting or inhibiting *de novo* fatty acid biogenesis (*ACACA, FASN, SCD5,* Figure 4A, C, and E), fatty acid uptake respectively activation (*SLC27A1, SLC27A2, SLC27A5, ACSL4, ACSL6,* Figure 4A, D, E), fatty acid elongation (*ELOVL4, ELOVL6, ELOVL7, TECR,* Figure 4B, D and E), and the intracellular transport of acyl-CoAs (*ACBD4, DBI, HACD1,* Figure 4B, C and E). In HepG2 cells, silymarin/silybin slightly increased ACC/*ACACA* (but not FASN) protein expression, which was significant for silybin (Figure S12B), while ACC phosphorylation, which inactivates ACC 120, tend to be decreased (Figure S12B). This weak stimulatory regulation of ACC by silymarin/silybin was not translated into increased cellular concentrations of i) malonyl-CoA (ACC product, Figure S12C), ii) long-chain fatty acids (FASN products, Figure S12C), or iii) long-chain acyl-CoAs (acyl-CoA synthetase products, Figure S12C). Conclusively, silybin and silymarin induce changes in fatty acid anabolism that may contribute to, but do not appear to be essential for, the lipid class switch from TGs to phospholipids.

### Silymarin/silybin A promotes phospholipid biosynthesis

To evaluate the effects of silymarin and silybin A on the biosynthesis of phospholipids and TGs, we treated HepG2 cells with silymarin or silybin A for 6 h and supplied them with ^13^C_2_, d_3_-labelled sodium acetate for additional 18 h. Newly synthesized PE and TG species were detected as M+3 signals by UPLC-MS/MS, with corrections applied for naturally occurring isotopes. As expected, both silymarin and silybin A significantly increased the incorporation of isotopically labelled acetate into PE species, particularly in PE(16:0_18:1) with M+3 in 16:0, PE(18:0_18:1) with M+3 in 18:0, and PE(18:0_18:1) with M+3 in 18:1 (Figure 4I and Figure S13A and C). Note that silymarin also led to a significant incorporation of labled acetate (M+3) into TG species (Figure 4J and Figure S13C and D) and that silybin A displayed a similar trend (Figure 4J and Figure S13C and D). These findings suggest that both silymarin and silybin A stimulate lipid biosynthesis, with silybin A showing a particular preference for phospholipids. Given that silybin, but not silymarin, reduces TG levels (Figure 1B, C), our data strongly suggests that silybin preferentially acts at the level of TG degradation and/or lipid droplet remodeling, an effect that may be compensated for silymarin by the stronger stimulatory effect on TG biosynthesis (Figure 4J).

### Silymarin but not silybin enhances fatty acid degradation for specific settings

Since the intracellular concentration of long-chain fatty acids is not markedly altered by silymarin/silybin (Figure S12C), while the fatty acid storage capacity in TGs is compromised (Figure 1B-D), we addressed the fate of fatty acids. On the one hand, they seem to be channeled towards phospholipid biosynthesis, as supported by our data (Figure 1B-D). On the other hand, they might be subjected to fatty acid oxidation *via* mitochondrial or peroxisomal pathways to sustain the energy demand for phospholipid biosynthesis 32,123-125. In support of this hypothesis, oral administration of silybin increased the mRNA expression of carnitine palmitoyl-transferase 1α (*Cpt1a*) in mouse liver, suggesting an efficient transfer of acyl-CoAs into mitochondria for β-oxidation 104. Transcriptomic analysis underlines that mitochondrial (*HADH, ACAT1, ACADVL*, Figure 4C, Figure S11) and peroxisomal β-oxidation (*ACOX3, HAO2*, Figure 4A) are enhanced for specific settings, and we confirmed in cultured HepG2 cells that silymarin increased the levels of the β-oxidation intermediate butyryl-CoA in cultured hepatocytes (Figure 4H). However, the effect does not seem to be mediated by silybin, which failed to enrich β-oxidation intermediates (Figure 4H). Since extensive fatty acid oxidation depletes fatty acid concentrations and thus competes with efficient phospholipid biosynthesis, we would expect fatty acid degradation to be kept in check. Consistent with these considerations, silymarin/silybin decreased the mitochondrial degradation of straight-chain, odd-chain, and branched fatty acids (*CPT2, ACAA1, ACAA2, HADH, ACADS, HADHB, PCCA, MCEE,* Figure 4B, C, D, E, Figure S11) as well as peroxisomal oxidation (*ABCD1, ACOX2, PHYH*, Figure 4C and E, Figure S11) and ketogenesis (*HMGCS2, BDH1, HMGCLL1,* Figure 4B, C, E), especially in mouse liver *in vivo* and Huh7.5.1 hepatoma cells *in vitro*. Fatty acid oxidation by CYP enzymes is also subject to intense regulation. Among the various CYP enzymes repressed by silymarin/silybin are those involved in the epoxidation and hydroxylation of polyunsaturated fatty acids (*CYP2C8, CYP2C9, CYP2C19, CYP3A4*, Figure 4E, Figure S11). ω-Oxidases are instead upregulated (*CYP4F2, CYP4A22*, Figure 4B and Figure S11), and results for *CYP1A1* are mixed (Figure 4B, C and E).

A detailed description of the impact of silymarin/silybin on cholesterol and CE metabolism is given in Supplementary Note 1.

Together, silymarin/silybin induce a lipid class switch from TGs to phospholipids by interfering with lipid metabolism at multiple nodes rather than strongly regulating a single specific target. Most importantly, silymarin/silybin limits TG biosynthesis and suppresses phospholipid degradation in both hepatocytes and extrahepatic cells, partly combined with enhanced phospholipid biosynthesis. These central adaptations are accompanied by pronounced changes in cholesterol and fatty acid metabolism.

### Efficacy of silybin in in vitro models of MAFLD and lipotoxicity

The predominant fatty acids present in TGs of the liver, both in healthy individuals and in MAFLD patients, are palmitic acid (PA, 16:0) and oleic acid (OA, 18:1) 126. Following previously published procedures 127, we established *in vitro* models of MAFLD and acute lipotoxicity by overloading human HepaRG cells (as a surrogate for normal hepatocytes 128) with a balanced saturated/unsaturated fatty acid mixture (PA:OA = 1:2) or by challenging them with the saturated fatty acid PA 127. We monitored the (time-dependent) increase in lipid droplets (Figure 5B and Figure S14A), TG levels (Figure 5C and Figure S14B), and phospholipid content, specifically PE (Figure 5D and Figure S14C) and PC levels (Figure S14D), and determined the consequences on cellular dehydrogenase activity (as a measure of cell viability) (Figure 5E and Figure S14E), viable cell number (Figure S14F), and membrane integrity (Figure S14G). PA/OA strongly increased lipid droplet staining (Figure 5B), elevated TG levels (Figure 5C), and caused a shift from PE (Figure 5D) to PC (Figure S14D) within 24 h. PA was less efficient in increasing TG levels and did not enhance the lipid droplet signal (Figure 5B), but raised the levels of both phospholipid subclasses investigated (Figure 5D, C and Figure S14D), as expected from the associated induction of ER stress and the UPR 129,130. The effects were less pronounced or even disappeared at longer incubation times (48 h) (Figure S14A-D). While OA/PA did not or hardly impair the metabolic activity of the cells (Figure 5E and Figure S14E), PA was cytotoxic within 24 h (EC_50_ = 70 µM) (Figure 5E, and Figure S14E), but did not yet disrupt membrane integrity (Figure S14G) or reduce the number of viable cells (Figure S14F).

To further validate the experimental model, we first investigated whether silybin A is able to induce a lipid class switch in unchallenged HepaRG cells, as expected from our studies in HepG2 cells (Figure 3). Indeed, silybin A (although the effects were less pronounced) induced a lipid class switch in HepaRG cells, but apparently from neutral lipids in lipid droplets to phospholipids (Figure 5B and Figure S14D), with little effect on total cellular TG levels (Figure 5C). We then investigated the effect of silybin A in the two disease models (PA or PA/OA treatment): silybin A still reduced the lipid droplet (but not TG) content (Figure 5B and C), but became less efficient in upregulating phospholipid levels (Figure 5D, and Figure S14C and D) and did not attenuate the lipotoxic drop in cell viability (Figure 5E). Our data indicate that silybin A preferentially redirects lipid metabolism from TGs to phospholipids in healthy hepatocytes and extrahepatic cells, and that this metabolic switch becomes less efficient under severe lipid overload, which might provide a mechanistic basis for the mixed results in clinical trials both, under disease and non-disease conditions 131-136.

### Activation of hepatic phase I and II metabolism in healthy mice

**Figure 6:**
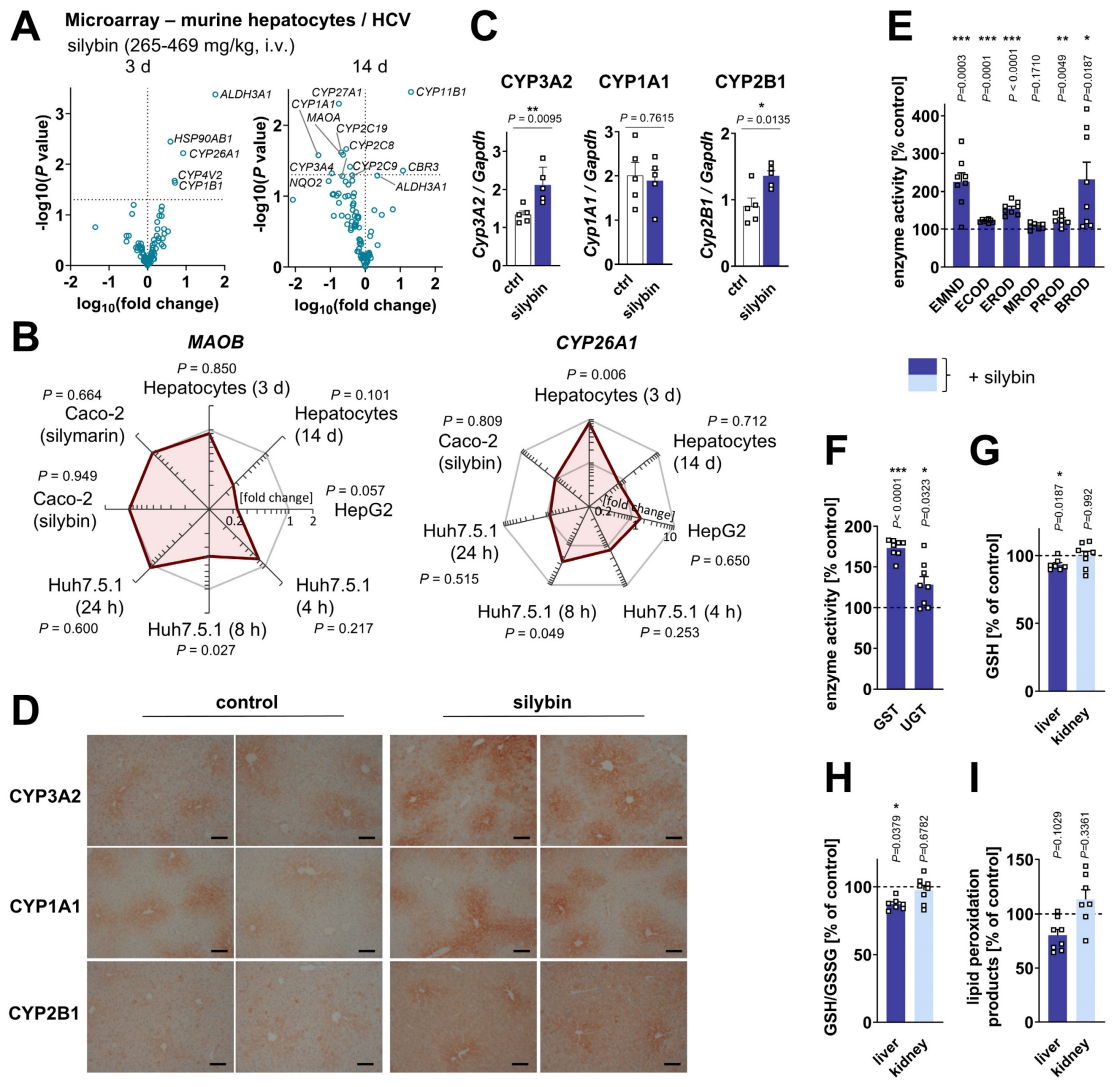
Elevated *CYP* enzyme expression and activity in mouse liver. (A) Comparative analysis of transcriptome data from hepatocytes derived from silybin-treated HCV-infected mice. Volcano plots compare the expression of lipid metabolic genes upon silybin treatment vs. vehicle control at day 3 and day 14. The dashed line indicates a non-adjusted *P*-value of 0.05; multiple two-tailed unpaired Student's *t*-tests. (B) Radar plots indicating the fold change in *MAOB* and *CYP26A1* expression by silybin relative to vehicle control. Non-adjusted *P* values given vs. vehicle control; multiple two-tailed unpaired Student's *t*-tests. (C-I) Mice received silybin hemisuccinate ('silybin'; 200 mg/kg, i.p.) or vehicle (0.9% NaCl) trice at 0, 12, and 24 h and were sacrificed after 37 h. (C) Protein expression of CYP3A2, CYP1A1, and CYP2B1 in mouse liver homogenates. Representative Western blots are shown in Figure S16. Individual values and mean + SEM; n = 5 mice/group. (D) Immunohistological analysis of CYP3A2, CYP1A1 and CYP2B1 expression in mouse liver; scale bar, 100 µm. n = 5 mice/group. (E) CYP activity measured by detecting the oxidative demethylation product formaldehyde (EMND) or the conversion of fluorogenic substrates in mouse liver homogenates. EMND: *N*-ethylmorphine *N*-demethylation (CYP3A), ECOD: 7-ethoxycoumarin *O*-deethylation (CYP1A and CYP2A-C), EROD: 7-ethoxyresorufin *O*-deethylation (CYP1A), MROD: 7-methoxyresorufin *O*-demethylation (CYP1A), PROD: 7-pentoxyresorufin *O*-depentylation (CYP2B), BROD: 7-benzyloxyresorufin *O*-debenzylation (CYP2A-C and CYP3A). Indicative CYP enzymes are listed in brackets. Individual values and mean + SEM; n = 8 mice/group. (F) Enzyme activity of GST and UGT in mouse liver homogenates. GST: gluthathione *S*-transferase; UGT: UDP-glucuronosyltransferase. Individual values and mean + SEM; n = 8 mice/group. (G-I) GSH levels (G), GSH/GSSG ratio (H) and lipid peroxidation (I) in mouse liver and kidney homogenates. Individual values and mean + SEM; n = 7 (liver GSH and GSH/GSSG, kidney lipid peroxidation) or n = 8 (kidney GSH and GSH/GSSG, liver lipid peroxidation) mice/group. *P < 0.05, **P < 0.01, ***P < 0.001 vs. control; two-tailed unpaired Student's *t*-test.

Silybin induces the expression of phase II enzymes (including glutathione S-transferase, GST) in mouse liver and other tissues 101,137-139. Instead, the consequences on CYP monooxygenases (phase I enzymes) are mixed 101,139-141, possibly due to superimposed direct enzyme inhibition, differences between healthy and diseased states, and different kinetics 142-145. To gain an overview about the global regulation of drug-metabolizing enzymes by silymarin/silybin, we analyzed the transcriptome data from the experimental systems described in section “*Silymarin/silybin acts on multiple nodes in the lipid metabolic network*” for changes in the expression of genes of the Reactome Pathway Database 109 categories 'metabolism - oxidation', 'phase I metabolism (compound functionalization)', and 'phase II metabolism (compound conjugation)'. Hepatocytes from silybin-treated HCV-infected mice showed a clear kinetic trend: genes of drug-metabolizing enzymes are initially upregulated (day 3) and then downregulated with prolonged treatment (day 14) (Figure 6A). Instead, the mRNA expression of *CYP* enzymes was differentially regulated in cell-based systems, with individual isoenzymes being up- or downregulated (Figure S15A-C), following independent kinetics (Figure S15B). With few exceptions (*CYP3A5, CYP26A1*, Figure 6A and B, Figure S15B), silymarin/silybin decreased the mRNA expression of those CYP enzymes that are prominently involved in drug metabolism (*CYP2B6, CYP2C8, CYP2C9, CYP2C19, CYP3A4*, Figure 6A and B, Figure S15B and D) and of amino oxidases (*AOC3, MAOA, MAOB*) (Figure 6A and B, Figure S15A and B), which oxidatively deaminate xenobiotic amines, in cell-based systems and *in vivo* after prolonged administration. Based on these data, we speculated that, in healthy mice receiving silybin for a short period of time (24 h), the increase in intracellular membranes is functionally coupled to membrane protein biosynthesis and accompanied by an increased availability of membrane-bound phase I and II isoenzymes that metabolize and detoxify xenobiotics 146,147. In fact, the protein levels of CYP3A2 and CYP2B1 (but not CYP1A1) were markedly enhanced in the liver of mice receiving silybin hemisuccinate, as shown by immunoblotting (Figure 6C and S16A) and visibly confirmed for all CYP isoforms tested by immunohistochemical analysis (Figure 6D). By contrast, the total amount of hepatic proteins decreased (Figure S16B). CYP enzyme expression was mainly concentrated around the endothelial cells of the central veins.

Accordingly, the biotransformation activity of CYP enzymes (Figure 6E), GST and UDP-glucuronosyltransferase (UGT) increased strongly (Figure 6F), as determined by the conversion of indicative substrates, possibly to support silymarin glucuronidation and excretion 36. Likely as a consequence of the increased GST turnover, the hepatic glutathione (GSH) pool decreased, with both GSH levels and the ratio to glutathione disulfide (GSSG) being significantly reduced (Figure 6G and H**)**. Since GSH, as an essential co-substrate of glutathione peroxidase (GPX)4, contributes to the reduction of lipid hydroperoxides and prevents degenerative cell death 148, we speculated that the decrease in GSH might enhance lipid peroxidation, which was, however, not the case (Figure 6I). Silybin actually attenuated the formation of lipid peroxidation products by trend in the liver but not in the kidney (Figure 6I), consistent with previous studies on silymarin/silybin 105,149,150. Together, the silybin-mediated accumulation of hepatic phospholipids (Figure 1B-D) is associated with an upregulation of membrane-bound detoxifying enzymes as well as GST isoenzymes that are present in different subcellular membrane compartments, including cytosol, mitochondria, ER, plasma membrane and nucleus 151.

For information on the effects of silymarin/silybin on vitamin A metabolism, see Supplementary Note 2.

## Discussion

**Figure 7:**
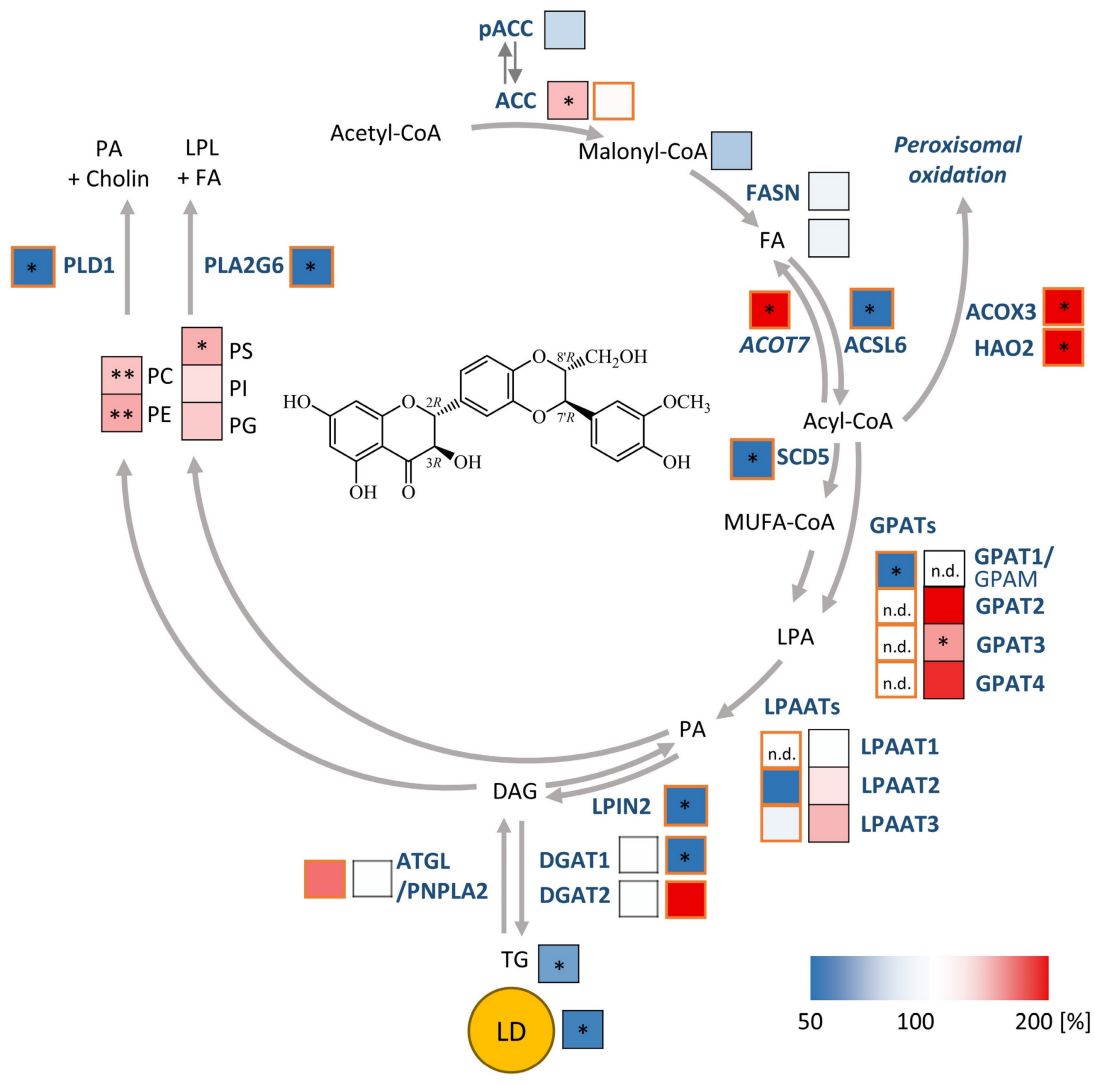
Proposed mechanisms of silymarin and its bioactive constituent silybin A in hepatocytes. Acetyl-CoA-carboxylase (ACC/ACACA) converts acetyl-CoA to malonyl-CoA, which is elongated to long-chain fatty acids by fatty acid synthase (FASN). Acyl-CoA esters are formed from free fatty acids (FAs) by acyl-CoA synthetases (ACSLs), which also activate exogenous fatty acids for further metabolism. Saturated acyl-CoAs are converted into monounsaturated acyl-CoAs (MUFA-CoA) preferentially by Δ9-desaturases, such as the stearoyl-CoA desaturase (SCD) isoenzyme 5. Acyl-CoA thioesterases (ACOTs) catalyze the opposite reaction, hydrolyzing acyl-CoAs to free fatty acids (FAs). Acyl-CoAs are used by glycerol-3-phosphate acyltransferases (GPATs) and lysophospholipid (LPL) acyltransferases/lysophosphatidic acid acyltransferases (LPLATs/LPAATs) to introduce fatty acyl-chains into the *sn*-1 and *sn*-2 positions of glycerol-3-phosphate and lysophosphatidic acid (LPA), respectively. The resulting PA is either converted to CDP-DAG for PI, PG, and PS biosynthesis or dephosphorylated to DAG for TG, PC, and PE biosynthesis by lipins (LPINs) and other PA phosphatases. LPIN2 also plays an important role in the regulation of fatty acid metabolism as nuclear transcriptional coactivator. Acylation of DAG by DGATs yields TGs, which are stored in lipid droplets and mobilized by ATGL/PNPLA2 and other triglyceride lipases, providing DAG and FAs. Phospholipid degradation is driven by a large number of phospholipases with different specificities. PLA2G6 releases saturated and unsaturated long-chain fatty acids from the *sn*-1 or *sn*-2 position of phospholipids, such as PC, PE and PA, whereas PLD1 specifically cleaves PC to PA and choline. By targeting multiple nodes, silymarin/silybin triggers a switch from TGs to phospholipids, thereby enriching intracellular membranes with phospholipids that have a balanced fatty acid composition. The increase in intracellular membranes is associated with enhanced membrane-associated biotransformation capacities. Mechanistically, silymarin/silybin inhibits phospholipid degradation, while moderately activating *de novo* phospholipid biosynthesis and stimulating TG catabolism in lipid droplets (LD), which in combination results in an effective channeling of TG-derived DAG and FAs into membrane biogenesis. In addition, silymarin induces the expression of genes involved in peroxisomal fatty acid degradation (HAO2, ACOX3), upregulates ACOT7, which hydrolyzes acyl-CoAs into FAs and CoA, and decreases the expression of ACSLs, that activate long-chain fatty acids. The color scale in the pathway diagram indicates the percentage changes in metabolite levels, lipid droplet counts, and enzyme expression by silybin relative to vehicle control in HepG2 cells (black bordered boxes) or by silymarin relative to vehicle control in HepG2 cells (orange bordered boxes). GPAM, glycerol-3-phosphate acyltransferase, mitochondrial.

The efficacy of silymarin and its major active component, silybin, in alleviating toxic liver injury and metabolic diseases 51 has been ascribed to hepatoprotective, anti-inflammatory, anti-oxidative response-inducing and membrane-stabilizing properties as well as to lipid-(TG and cholesterol)-lowering effects 38,152,153. Here, we report that silybin induces a metabolic switch in hepatocytes and extrahepatic cells, especially under non- or pre-disease conditions, linking hepatic TG metabolism with membrane biogenesis and potentially biotransformation activity (Figure 7), with the latter potentially contributing to the liver protective function.

### Effects of silymarin/silybin on lipid-metabolizing enzymes

Specifically, silybin treatment lowers TG levels, while limiting phospholipid degradation in hepatocytes and, under certain settings, additionally stimulates phospholipid biosynthesis, reflecting a net transfer of fatty acids from TGs to phospholipids (Figure 7). Context-dependent adaptations of fatty acid biosynthesis, intracellular transport, mitochondrial and peroxisomal degradation, cholesterol biosynthesis, and sterol metabolism further add to the class switch from TGs to phospholipids. Consequently, the size of lipid droplet decreases while the content of membrane phospholipids increases. At the same time, intracellular membranes are formed that, when coordinated with an upregulation of phase I and II membrane-(associated) enzymes, may enhance the biotransformation capacity of subcellular compartments, such as the ER. A decrease of hepatic lipid droplet size and TG levels is generally considered beneficial in metabolic diseases 10,154. In support of this principle, increased ATGL/PNPLA2 expression protects against hepatic steatosis 155, whereas ATGL/PNPLA2 repression promotes the development of MAFLD 156,157. On the other hand, lipolysis is also associated with elevated levels of free fatty acids, which in excess may be lipotoxic to hepatocytes or cause oxidative stress when being degraded by mitochondrial or peroxisomal β-oxidation 6,158. Thus, suppression of TG biosynthesis by selective inhibition of DGAT2 improves steatohepatitis and insulin sensitivity, but at the same time exacerbates liver damage in a methionine and choline deficient (MCD) mouse model of NASH 6. However, in alternative animal models (such as those using diets high in fructose, saturated fat, and cholesterol 159, or Western diets 160), DGAT2 inhibition reduced steatosis without affecting inflammation or fibrosis in the latter. Conclusively, the reduction of lipid droplets and TGs alone does not fully explain the hepatoprotective function of silymarin and silybin, but requires an efficient channeling of the degradation products into non-toxic metabolites, i.e., phospholipids, as suggested by our results. A similar redistribution was observed for the inhibition of DGAT2 (PF-06424439), although it was restricted to the ER and PE species 161. This metabolic switch to phospholipids is likely to be of biomedical relevance in toxic liver injury and MAFLD/MASH, where either the hepatic content of total phospholipids or specific phospholipid subclasses is decreased 35,53,54,62. Consistent with this metabolic dysregulation, many key regulatory factors of MAFLD involve enzymes that are central to phospholipid and TG metabolism, including PNPLA3 30, LPIAT1/MBOAT7 19,29,30,131,162, ATGL/PNPLA2 22, iPLA2/PLA2G6 23,24, PLA2G7 163, PLA2 activity of PRDX6 27, PLD1 28, and LPIN2 21. Altogether, genetic variations or changes in protein expression of these enzymes define the risk of developing MAFLD 20,164 and, together with other regulatory mechanisms, may shape the aberrant lipid composition of the diseased liver, with decreasing phospholipids and increasing TGs 32-35,165-167*.* Silymarin/silybin regulates a significant number of these lipid metabolic genes, including *iPLA2/PLA2G6*, *ATGL/PNPLA2, PLD1*, *LPIN2*, and by trend* PNPLA3* (which is a major genetic risk factor for MAFLD 131) and *HSD17B13*, counteracting the observed dysregulation in liver diseases. In line with our findings, a recently published randomized controlled trial showed that silybin treatment improved MAFLD parameters only in patients without a genetic predisposition, while it was ineffective in patients carrying either one or a combination of mutations responsible for genetically inherited forms of MAFLD 131. Given that the metabolic and genetic components of MAFLD differ fundamentally 168, these findings suggest that silymarin/silybin may be particularly effective against the metabolic, but not against the genetic, form of MAFLD.

In addition, we show here that silymarin/silybin represses the potentially disease-promoting oxidative metabolism of fatty acids (via mitochondrial and peroxisomal pathways but also CYP enzymes) in many settings, including primary mouse hepatocytes, although opposite regulations were also observed at the transcriptome level.

Diverse mechanisms have been discussed for the TG- and cholesterol-lowering activity of silymarin/silybin: i) reduced lipid resorption, ii) upregulated cholesterol efflux *via* (ABC) transporters that excrete cholesterol from the liver to the bile 104,152,169, and iii) adjustments in lipid biosynthesis, transport, and degradation by targeting major transcription factors in lipid metabolism 58,61,104,170-172, such as PPARα/PPARγ, LXR; ChREBP and SREBP-1c 55,171-174. Lipid-metabolic proteins proposed to be affected by silymarin/silybin include enzymes involved in fatty acid biosynthesis (ACC/*ACACA*, FASN, SCD-1), uptake (FABP5), and degradation (CPT1α, ACOX), phospholipid biosynthesis (GPATs), lipid transport (MTTP), and phosphatidic acid/TG turnover (PNPLA3) 58,61,104,170-172,175. While these studies focused on the ability of silymarin/silybin to restore expression levels under pathophysiological conditions, we first addressed non-stressed cells, healthy mice, and pre-disease conditions. Comparative transcriptomic analyses in four different model systems confirmed that silymarin/silybin differentially regulates pathways contributing to TG and phospholipid metabolism. Specifically, our analysis revealed that silybin broadly manipulates phospholipid metabolism, although the exact mechanism varies between model systems and experimental settings. Overall, we show that silymarin/silybin reduces phospholipid degradation by repressing various phospholipases (*PLA2G1B*, *PLA2G6*, *PLD1,* and/or *PLD6*) or inducing the expression of phospholipase-suppressing factors (e.g., *PLA2R1*). However, the specific enzymes targeted can vary between different experimental models, and in some cases alternative enzymes may be upregulated, potentially acting as compensatory mechanisms. Less consistently, silymarin/silybin also upregulates the expression of factors involved in acyl-CoA supply (e.g., *FASN*, *SLC27A1, ELOVL7, ACSL4)* and phospholipid biosynthesis (e.g., *MBOAT2*, *LPGAT1*), *via* both *de novo* and remodeling pathways. In support of the relevance of this mechanism, we show that silymarin and silybin increase the incorporation of isotopically labeled acetate into phospholipids. On the other hand, silymarin/silybin seems to suppress TG biosynthesis in several experimental systems, mainly by repressing enzymes involved in the generation of DAGs and their acylation to TGs (e.g. via DGAT1 and DGAT2). In HepG2 cells, DGAT1/2 protein expression was not repressed by either silybin or silymarin (at least under our experimental conditions), allowing us to determine independent effects on the rate of TG biosynthesis, which actually increased, especially for silymarin. These data suggest that silymarin/silybin rather than suppressing TG biosynthesis regulates TG remodeling in HepG2 cells, as supported by the observed decrease in lipid droplet size. The situation may be different in other experimental systems, in which suppressive effects on TG biosynthesis are expected based on transcriptome analysis.

The important role of silymarin/silybin in modulating lipid metabolism has been recognized before, and effects on ACC 55,108,176, FASN 55,108,169,172,176-178, SCD-1 14,104,172,179, GPATs 104, PNPLA3 104,175,177 , FABP5 104,172,179, CPT1a 55,104,108,172,178,180, MTTP 104, ACOX 104, PPARα/PPARγ 55,104,172,173,175,177,180,181 and SREBP-1c 55,104,175-177,180,181 have been reported independently, either under disease 55,60,61,104,150,171,175,176,182 or non-disease conditions 58,169,177,178, largely without considering their interplay. By converging transcriptomics, metabololipidomics, and functional studies, we put these individual findings into context. Thus, our data strongly suggest that silybin, by coordinating multiple enzymes involved in lipid metabolism, facilitates the efficient channeling of fatty acids from TGs into phospholipids unless cells experience extensive lipid overload, with potential implications for disease prevention. The liver and body weight of the treated mice were reduced accordingly. We conclude that silybin buffers excessive hepatic TG accumulation, a hallmark of MAFLD, and redirects fatty acids by limiting phospholipid degradation and stimulating (energy-consuming) phospholipid biosynthesis and possibly membrane biogenesis.

Gavage of silymarin/silybin to mice reduced pathological changes in liver and serum lipid composition 54,62,63,183,184, and its beneficial effects were anticipated to depend on either a decreased cholesterol/phospholipid ratio 62, a reduced proportion of SM relative to PC 62,183, or increased PE levels 63. Our lipidomic analysis essentially confirmed an efficient upregulation of PE and other glycerophospholipids (rather than sphingolipids) in mouse liver by silybin. The influence on membrane properties is difficult to assess, but the homogeneous upregulation of phospholipid classes suggests that there are no major changes. It should be noted that we did not analyze free cholesterol, a major membrane component that affects rigidity and fluidity 185.

### Structural aspects of silybin A for the induction of a metabolic switch

Structure-activity relationship studies underscore that silybin functionally intervenes at two (or more) different sites in lipid metabolism to accomplish the shift from TGs to phospholipids. While the saturation of the flavonoid scaffold at the 2,3-position is essential for phospholipid accumulation (but has little effect on the amount of cellular TG), changes in the stereochemistry at the dioxan ring reduced both the effect on phospholipid and TG levels. The introduction of a 2,3-double bond yielding 2,3-dehydrosilybin even resulted in a decrease of the PS and PG content. The biosynthesis of these acidic phospholipids requires the conversion of DAG to phosphatidic acid, whereas PC and PE can be synthesized directly from DAG (Figure 7) 186. Interestingly, the ring rearrangement in the hemiacetal did not substantially hamper the activity on phospholipids or TGs when compared to the diastereomeric silybin mixture. Together, silybin modulates phospholipid and TG metabolism through independent pathways, with the magnitude and directionality of the effect strongly dependent on the stereochemistry and saturation of the flavonolignan. Consistent with the hypothesis that silybin upregulates the intracellular phospholipid content also independently of the decrease in TGs, pharmacological inhibition of specific isoenzymes involved in lipid droplet degradation (ATGL) or lipid droplet biogenesis (DGAT2) did not markedly alter the cellular phospholipid content.

### Impact of silymarin/silybin on drug-metabolizing enzymes

We also show that silymarin/silybin increases the content of phospholipids in hepatocytes, thereby forming intracellular membranes that are likely to host enzymes involved in biotransformation. On the one hand, phase I and phase II enzymes provide protection against multiple xenobiotics and diminish drug-induced hepatotoxicity 187. On the other hand, phase I CYP enzymes convert various xenobiotics, e.g., the analgesic drug acetaminophen (paracetamol), into toxic metabolites 188. Silymarin has been proposed to protect against toxic liver injury i) by inhibiting CYP enzymes and suppressing deleterious metabolism, ii) by inducing the expression of phase II enzymes such as UGT and GST, and iii) by upregulating membrane transporters that enhance the excretion of xenobiotics 137,138. While our data confirm an upregulation of phase II enzyme activities by silybin, we found that not only the expression but also the activity of multiple CYP isoenzymes was increased rather than decreased under short-term treatment. We suggest that the mixed outcomes of studies investigating the effect of silymarin/silybin on CYP enzymes originate from kinetic regulation and the competition between CYP expression and inhibition, which seems to be sensitive to the dosage, route of application, formulation, and/or duration of treatment 101,137,139. Thus, the elevated CYP enzyme activity in our experimental design is likely due to an increased CYP protein expression that masks the inhibitory effect of silybin on CYP activity. In support of this hypothesis, silymarin administration to rats increased the hepatic cytochrome P450 levels with defined kinetics 189, as further validated here at the transcriptome level in HCV-infected mice treated with silybin. Overall, silymarin/silybin induced a rapid upregulation of drug-metabolizing enzymes, followed by a decrease in expression with prolonged treatment. Another factor that may contribute to the variable study results on CYP enzymes is that healthy and diseased tissues seem to respond differently to silybin. In fact, silymarin/silybin partially restores CYP enzyme homeostasis under pathophysiological conditions 141,142,144, whereas effects in healthy individuals are more diverse 140,145,152. It should be noted that, with a few exceptions, most *in vivo* animal and human studies have failed to confirm that silymarin/silybin substantially interferes with the pharmacokinetics of various drugs that are metabolized by CYP enzymes 36. However, one of the few studies showing significant effects in healthy volunteers found, consistent with our results in mice, that silymarin (140 mg, daily) increased the clearance and decreased the C_max_ values of metronidazole, a drug that is metabolized by CYP3A4 and CYP2C9 190. Whether the modulation of CYP enzyme activity by silymarin is of clinical relevance in patients with MAFLD remains elusive and needs to be systematically evaluated for specific formulations, dosages and CYP isoenzymes in future kinetic studies.

### Efficacy of silymarin/silybin in the treatment of MAFLD

Given the mixed results of silymarin/silybin on lipid metabolism in health and disease 131-136, we investigated the ability of silybin to redirect lipid metabolism in *in vitro* models of MAFLD (achieved by massive fatty acid overload) and acute lipotoxicity (induced by excess saturated fatty acids). On the one hand, silybin A consistently reduced lipid droplet content below basal levels in unstressed and fatty acid-challenged hepatocytes, notably superior to selective inhibitors of DGAT1 or DGAT2 or antagonist of PPARγ. On the other hand, silybin A was considerably less effective in redirecting fatty acids from lipid droplets to phospholipids in stressed as compared to non-stressed cells and did not protect against lipotoxicity. Our results suggest that the silymarin/silybin-induced lipid class switch from TGs to phospholipids is particularly effective in protecting against adverse dysregulation of lipid metabolism in normal and pre-disease conditions, whereas the beneficial effects appear to be largely limited to reducing TGs in liver diseases associated with severe TG accumulation, such as MAFLD. Further studies are needed to elucidate the general relevance of this dual mechanism by directly comparing healthy and diseased states in clinical trials.

### Differences between the effects of silymarin and silybin

Silymarin and silybin modulate lipid metabolism in hepatocytes in a comparable manner, but there are also substantial differences, and some effects are seen only for silymarin or silybin.

First, only silymarin elevated the levels of butyryl-CoA, an intermediate of β-oxidation (1.5-fold), implying that fatty acid degradation is stimulated by components of silymarin other than silybin. Whether such polypharmacological modulation by silymarin has advantages, is poorly understood. On the one hand, we discussed above that excessive β-oxidation under stress conditions could be detrimental due to the production of reactive oxygen species (ROS)^14^. On the other hand, the induction of fatty acid catabolism by silymarin is moderate and could help to meet the energy demands for stress-adaptive, regenerative pathways (including lipid remodeling), especially since the antioxidants in silymarin already counteract ROS accumulation 191.

Second, we observed significant differences in the effect of silymarin and silybin on triglyceride levels in different cell types. Whereas silybin significantly reduces TG levels in hepatocytes and has no effect in monocytes, silymarin hardly affects TG levels in hepatocytes but robustly decreases them in monocytes. While the accumulation of TGs in hepatocytes is critical for the development of MAFLD, the extent to which reducing TGs in monocytes benefits the disease process is not well understood.

Third, silymarin and silybin clearly differ in how they manipulate the expression of lipid-metabolizing enzymes, although some of the effects may be related to the use of different model systems, which is a limitation of this study. For example, the expression of DGAT2 is significantly downregulated only in hepatocytes isolated from silybin-treated mice, without effects in silymarin-treated HepG2 and Huh7.5.1 liver cells. However, reduced expression of DGAT1 is observed in both hepatocytes from silybin-treated mice and silymarin-treated HepG2 and Huh7.5.1 cells, suggesting that the repression of DGAT2 is silybin-specific. Additional differential effects are seen in the regulation of phospholipases: PLA2G1B is downregulated in hepatocytes from silybin-treated mice, whereas PLD1 is downregulated in silymarin-treated HepG2 and Huh7.5.1 cells.

In addition to modulating lipid metabolism, silymarin is known to have beneficially effects on other pathogenic mechanisms associated with MAFLD, such as inflammation and glucose metabolism 192, the latter of which is also supported by our data.

## Conclusion

The milk thistle extract silymarin and its bioactive component silybin have unique lipid-modulating properties. Rather than targeting one particular pathway, silymarin/silybin affects lipid metabolism at multiple hubs. In hepatic pre-disease states, silybin decreases TG content, while attenuating phospholipid degradation and stimulating phospholipid biosynthesis. In combination with the parallel reprogramming of phase I and II metabolism, this lipid class switch seems to expand functional intracellular membranes and redirects the hepatic biotransformation capacity. Considering that the selective inhibition of TG biosynthesis actually enhances liver damage, as suggested by preclinical studies with DGAT2 antisense oligonucleotide treatment 6, we propose that the lipid metabolic switch from TGs to phospholipids in hepatocytes and potentially other liver cells (i.e., Kupffer cells) and extrahepatic cells critically contributes to the liver-protective (rather than disease-alleviating) function of silybin. The beneficial reprogramming of lipid metabolism is based on the absolute configuration of silybin as well as the saturation of ring C in the flavonoid scaffold at the 2,3-position. These structural aspects contribute differentially to the TG-lowering and phospholipid-accumulating activities of silybin, and structural modifications realized in minor components of milk thistle allow dissection of both activities.

In conclusion, our results shed light on the mechanisms underlying liver protection by silymarin/silybin under physiological and pathophysiological conditions. Although silymarin/silybin appears to have the potential to improve the biochemical hallmarks of MAFLD and may be beneficial in mild forms of the disease through the mechanisms proposed here, our data suggest that this mechanism may be less effective or even ineffective in severe disease states. Whether silymarin/silybin exerts beneficial effects under these conditions when combined with adjunctive supplements or dietary restriction remains elusive. Future evidence-based studies with a larger number of participants and longer follow-up are needed to explore the relevance of the silybin A-induced lipid metabolic switch in humans, both for disease prevention and under pathophysiological conditions.

## Supplementary Material

Supplementary figures and notes.

## Figures and Tables

**Table 1:** Primer sequences used in real-time quantitative PCR experiments

gene	sense primer (5' → 3')	anti-sense primer (5' → 3')
hGPAT1	GAAGCTGGAGCTGCTGGGCA	AAAGCCACACTCACCCCATTCCT
hGPAT2	TCGTGCTGGGCCAATGTACTG	AGGAGAACTCCCCCAGGAGC
hGPAT3	CTGCCAGACAGCAGCCTCAA	GCCATGAACCTGGCCAACCA
hGPAT4	GCCGCTCAGGATGCACTGG	CCGTGCACTTGACCCACCAT
hLPLAT1/hLPAAT1	GAGACACAGCCATCCGCCAC	GCAAGATCTTCATGTTCTCGACGTT
hLPLAT2/hLPAAT2	CGCAACGACAATGGGGACCT	TGCACTGTGACTGTTCCTGAAGT
hLPLAT3/hLPAAT3	CGGCTGCAGGCTTGTCCA	CAGTTGAGGCGGCGGTGAG
hß-Actin	ACAGAGCCTCGCCTTTGCC	CCATCACGCCCTGGTGCC
hGAPDH	TTTGCGTCGCCAGCCGAG	TTCTCAGCCTTGACGGTGCC

## Abbreviations

ACC: acetyl-CoA carboxylase
ACOX3: acyl-CoA oxidase 3, pristanoyl
ACOT7: acyl-CoA thioesterase 7
ACSL6: acyl-CoA synthetase long chain family member 6
ATF6: activating transcription factor
ATGL: adipocyte triglyceride lipase
BSA: bovine serum albumin
CE: cholesteryl esters
CL: chemiluminescence
CPT1a: carnitine palmitoyl-transferase 1α
CYP: cytochrome P_450_
DAG: diacylglycerol
DGAT: diacylglycerol-O-acyltransferase
ER: endoplasmic reticulum
FASN: fatty acid synthase
GPAM: glycerol-3-phosphate acyltransferase, mitochondrial
GPAT: glycerophosphate acyltransferase
GPX: glutathione peroxidase
GSH: glutathione
GSSG: glutathione disulfide
GST: glutathione S-transferase
HAO2: hydroxyacid oxidase 2
HCV: hepatitis C virus
HSD17B13: 17-beta-hydroxysteroid dehydrogenase 13
LPIAT1: lysophosphatidylinositol acyltransferase 1
LPIN2: Lipin 2
LPAAT: lysophosphatidic acid acyltransferase
LPLAT: lysophospholipid acyltransferase
MBOAT7: membrane-bound O-acyltransferase domain-containing 7
MAFLD: metabolic dysfunction-associated steatotic liver disease
MASH: metabolic dysfunction-associated steatohepatitis
NAFLD: non-alcoholic fatty liver disease
NASH: non-alcoholic steatohepatitis
PC: phosphatidylcholine
PE: phosphatidylethanolamine
PG: phosphatidylglycerol
PI: phosphatidylinositol
PNPLA: patatin-like phospholipase domain-containing
PLA1A: phospholipases A1
PLA2: phospholipase A2
iPLA2/PLA2G6: calcium-independent phospholipase A2 / phospholipase A2 Group VI
PLA2G7: phospholipase A2 Group VII
PLD1: phospholipase D1
PRDX6: peroxiredoxin 6
PS: phosphatidylserine
ROS: reactive oxygen species
SCD5: stearoyl-CoA desaturase 5
SM: sphingomyelin
SREBF: sterol regulatory element binding transcription factor 1
TG: triglyceride
UGT: UDP-glucuronosyltransferase
UPR: unfolded protein response
XBP1s: spliced form of X-box-binding protein 1
