# Supplementary material for: Silybin A from Silybum marianum reprograms lipid metabolism to induce a cell fate-dependent class switch from triglycerides to phospholipids
Source: Theranostics. 2025 Jan 6;15(5):2006–34. doi: 10.7150/thno.99562 (PMC11780512; doi:10.7150/thno.99562)

Supporting Information for

ORIGINAL ARTICLE

Silybin A from *Silybum marianum* reprograms lipid metabolism to induce a cell fate-dependent class switch from triglycerides to phospholipids

Solveigh C. Koeberle[a,b†,*], Maria Thürmer[c,†], Fengting Su[a,b], Markus Werner[c], Julia Grander[b], Laura Hofer[b], André Gollowitzer[b], Loc Le Xuan[b], Felix J. Benscheid[b], Ehsan Bonyadi Rad[b], Armando Zarrelli[d], Giovanni Di Fabio[d], Oliver Werz[c], Valeria Romanucci[d], Amelie Lupp[e], Andreas Koeberle[a,b,c*]

* Corresponding authors: Andreas Koeberle, University of Graz, Graz, 8010, Austria.

Solveigh C. Koeberle, University of Graz, Graz, 8010, Austria.

[a]*Institute of Pharmaceutical Sciences/Pharmacognosy University of Graz, 8010 Graz, Austria*
[b]*Michael Popp Institute and Center for Molecular Biosciences Innsbruck (CMBI), University of Innsbruck, 6020 Innsbruck, Austria*
[c]*Department of Pharmaceutical/Medicinal Chemistry, Institute of Pharmacy, Friedrich Schiller University Jena, 07743 Jena, Germany*
[d]*Department of Chemical Sciences, University of Napoli Federico II, Naples, Italy*
[e]*Institute of Pharmacology and Toxicology, Jena University Hospital, Jena, Germany*

*Corresponding authors. +43 316 380 - 8630.

E-mail addresses: andreas.koeberle@uni-graz.at (Andreas Koeberle).

solveigh.koeberle@uni-graz.at (Solveigh Koeberle).

Supplementary Figures

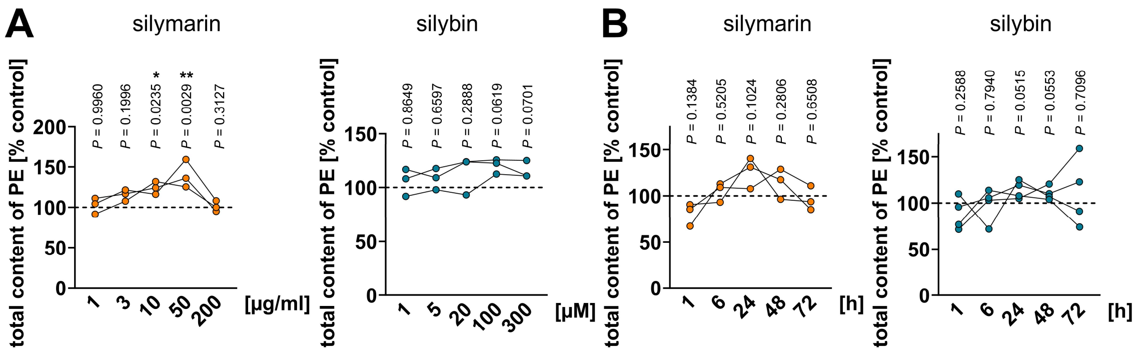

Figure S1. Concentration- and time-dependent effects of silymarin and silybin on cellular PE levels. (A, B) HepG2 cells were treated with silymarin, silybin or vehicle (ethanol for silymarin, DMSO for silybin) at the indicated concentrations for 24 h (A) or with silymarin (10 µg/ml), silybin (20 µM) or vehicle (ethanol for silymarin, DMSO for silybin) for the indicated incubation times (B). Independent datasets connected by lines; n = 3 (A, B, silymarin) or n = 4 (B, silybin). $^{*}P < 0.05$, $^{**}P < 0.01$ vs. vehicle control for the respective time point; two-tailed paired Student's *t*-test.

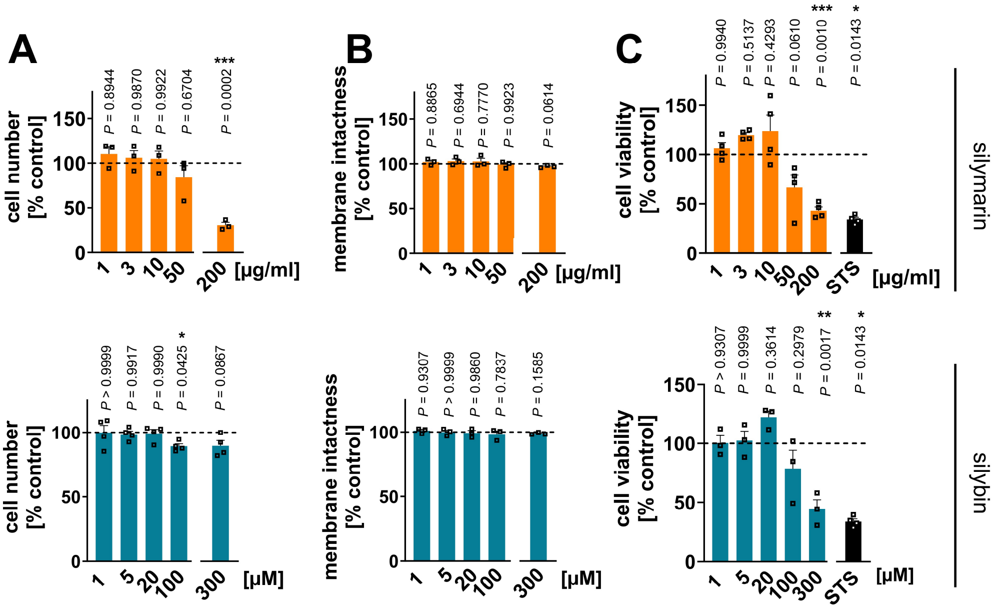

Figure S2. Effects of silymarin and silybin on cell number, membrane integrity and cell viability. HepG2 cells were treated with silymarin and silybin (at the indicated concentrations), staurosporine (STS, 1 µM) (C), or vehicle (ethanol for silymarin, DMSO for silybin and STS) for 24 h. (A) Cell numbers. Individual values and mean + SEM; n = 3 (silymarin), or n = 4 (silybin). (B) Membrane intactness measured by trypan blue staining. Individual values and mean + SEM; n = 3; effects of silymarin (200 µg/ml) and silybin (300 µM) were assessed in independent experiments (A, B). (C) Cell viability determined by MTT assay. Individual values and mean + SEM; n = 3 (silybin) or n = 4 (silymarin and STS). *$P < 0.05$, **$P < 0.01$, ***$P < 0.001$ vs. vehicle control; ordinary one-way ANOVA + Tukey HSD *post hoc* tests (C) and two-tailed paired Student's *t*-test (A and B, 200 µg/ml silymarin and 300 µM silybin, C, STS).

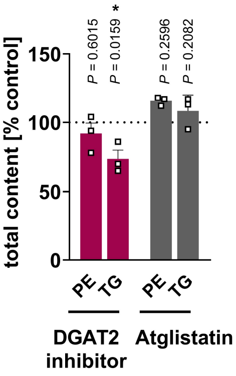

Figure S3. Influence of DGAT and ATGL inhibition on the phospholipid and TG content of hepatocytes. HepG2 cells were treated with the DGAT inhibitor PF 06424439 or the ATGL inhibitor atglistatin for 24-30 h. Total amounts of PE and TG were determined by UPLC-MS/MS. Individual values and mean + SEM; n = 3. *$P < 0.05$ vs. vehicle control; two-tailed unpaired Student's *t*-test.

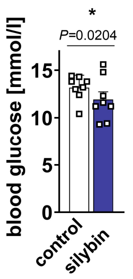

Figure S4. Blood glucose levels. Mice received silybin hemisuccinate (‘silybin’; 200 mg/kg, i.p.) or vehicle (0.9% NaCl) trice at 0, 12, and 24 h and were sacrificed after 37 h. Individual values and mean from n = 8 mice/group, *$P < 0.05$ vs. vehicle control; two-tailed unpaired Student’s *t*-test.

A

monocytes

PC

	control	silymarin	control	silybin
PC(16:0_16:0)	1.217 ± 0.397	1.485 ± 0.345	0.972 ± 0.06	1.053 ± 0.082
PC(16:0_18:1)	0.681 ± 0.254	1.089 ± 0.276	0.456 ± 0.015	0.542 ± 0.019
PC(18:0_18:1)	0.303 ± 0.099	0.45 ± 0.06	1.804 ± 0.069	2.064 ± 0.116
PC(18:1_18:1)	0.329 ± 0.133	0.518 ± 0.152	0.374 ± 0.035	0.440 ± 0.035
PC(18:1_20:1)	0.053 ± 0.023	0.08 ± 0.02	0.081 ± 0.016	0.101 ± 0.016
PC(16:0_18:2)	0.098 ± 0.029	0.211 ± 0.034	0.178 ± 0.007	0.232 ± 0.01
PC(18:0_18:2)	0.106 ± 0.039	0.188 ± 0.017	0.51 ± 0.01	0.566 ± 0.029
PC(16:0_20:3)	0.045 ± 0.019	0.06 ± 0.028	0.038 ± 0.004	0.046 ± 0.004
PC(16:0_20:4)	0.094 ± 0.032	0.164 ± 0.039	0.232 ± 0.008	0.248 ± 0.006
PC(18:1_20:4)	0.037 ± 0.012	0.064 ± 0.013	0.093 ± 0.003	0.105 ± 0.004
PC(18:0_20:3)	0.011 ± 0.004	0.017 ± 0.005	0.019 ± 0.002	0.048 ± 0.021
PC(18:0_20:4)	0.012 ± 0.003	0.034 ± 0.004	0.028 ± 0.001	0.024 ± 0.000
PC(16:0_22:5)	0.291 ± 0.078	0.347 ± 0.073	0.213 ± 0.025	0.226 ± 0.022
PC(18:0_22:6)	0.058 ± 0.016	0.078 ± 0.008	0.021 ± 0.002	0.021 ± 0
PC 32:0 E (16:0)	0.02 ± 0.008	0.023 ± 0.001	0.183 ± 0.02	0.182 ± 0.017
PC 34:2 E (18:2)	0.128 ± 0.047	0.197 ± 0.046	0.255 ± 0.013	0.257 ± 0.009

PE

	control	silymarin	control	silybin
PE(16:0_18:1)	0.057 ± 0.02	0.102 ± 0.031	0.148 ± 0.019	0.24 ± 0.031
PE(18:0_18:1)	0.693 ± 0.289	0.978 ± 0.28	1.261 ± 0.067	1.323 ± 0.035
PE(18:1_18:1)	0.156 ± 0.058	0.263 ± 0.087	0.204 ± 0.024	0.322 ± 0.045
PE(18:1_20:1)	0.039 ± 0.017	0.076 ± 0.03	0.026 ± 0.002	0.035 ± 0.003
PE(18:0_20:4)	0.483 ± 0.174	0.756 ± 0.206	1.781 ± 0.112	2.134 ± 0.142
PE(18:0_22:5)	0.046 ± 0.02	0.069 ± 0.022	0.128 ± 0.007	0.145 ± 0.008
PE 34:1 E (16:0)	0.158 ± 0.051	0.193 ± 0.053	0.247 ± 0.032	0.281 ± 0.026

PS

	control	silymarin	control	silybin
PS(18:0_18:1)	0.373 ± 0.127	0.535 ± 0.151	0.636 ± 0.036	0.726 ± 0.052
PS(18:0_18:2)	0.038 ± 0.013	0.053 ± 0.015	0.086 ± 0.006	0.103 ± 0.005
PS(18:0_20:3)	0.125 ± 0.053	0.174 ± 0.061	0.081 ± 0.008	0.093 ± 0.012
PS(18:0_20:4)	0.053 ± 0.009	0.104 ± 0.029	0.338 ± 0.044	0.442 ± 0.068

PI

	control	silymarin	control	silybin
PI(18:0_18:1)	0.062 ± 0.021	0.093 ± 0.029	0.032 ± 0.002	0.032 ± 0.002
PI(18:0_18:2)	0.037 ± 0.007	0.072 ± 0.021	0.024 ± 0.000	0.024 ± 0.001
PI(18:0_20:4)	0.482 ± 0.144	0.818 ± 0.268	0.293 ± 0.011	0.334 ± 0.015
PI(18:0_20:3)	0.029 ± 0.012	0.045 ± 0.015	0.045 ± 0.003	0.052 ± 0.003

PG

	control	silymarin	control	silybin
PG(14:0_18:1)	0.046 ± 0.024	0.055 ± 0.026	0.041 ± 0.006	0.088 ± 0.016
PG(16:0_18:1)	0.010 ± 0.003	0.038 ± 0.007	0.025 ± 0.003	0.021 ± 0.003
PG(18:0_18:1)	0.014 ± 0.002	0.044 ± 0.010	0.008 ± 0.001	0.01 0 ± 0.001

SM

	control	silymarin	control	silybin
14:0 SM	0.039 ± 0.011	0.059 ± 0.01	0.022 ± 0.002	0.025 ± 0.001
16:0 SM	1.161 ± 0.383	1.667 ± 0.405	0.602 ± 0.019	0.630 ± 0.023
18:0 SM	0.061 ± 0.019	0.078 ± 0.018	0.033 ± 0.002	0.041 ± 0.003

TG

	control	silymarin	control	silybin
16:0_16:0_18:1	0.002 ± 0.000	0.002 ± 0.000	0.001 ± 0.000	0.001 ± 0.000
16:0_18:1_18:1	0.009 ± 0.002	0.006 ± 0.001	0.005 ± 0.001	0.006 ± 0.001
18:0_18:1_18:1	0.008 ± 0.001	0.005 ± 0.001	0.006 ± 0.001	0.006 ± 0.001

B

HepG2

PC

	control	silymarin	control	silybin
PC(16:0_16:0)	0.762 ± 0.058	0.943 ± 0.057	0.714 ± 0.083	0.778 ± 0.079
PC(16:0_16:1)	1.369 ± 0.125	2.123 ± 0.056	1.082 ± 0.164	1.257 ± 0.173
PC(16:0_18:1)	3.377 ± 0.246	4.94 ± 0.123	2.97 ± 0.414	3.486 ± 0.441
PC(18:1_18:1)	2.149 ± 0.17	3.275 ± 0.048	2.492 ± 0.264	3.03 ± 0.194
PC(16:0_18:2)	0.451 ± 0.044	0.698 ± 0.024	0.47 ± 0.063	0.523 ± 0.052

PE

	control	silymarin	control	silybin
PE(16:0_16:1)	0.155 ± 0.013	0.255 ± 0.011	0.196 ± 0.01	0.228 ± 0.003
PE(16:0_18:1)	0.67 ± 0.069	1.129 ± 0.044	1.545 ± 0.764	1.85 ± 0.831
PE(18:0_18:1)	1.029 ± 0.128	1.735 ± 0.11	4.748 ± 1.2	6.695 ± 1.636
PE(18:1_18:1)	1.022 ± 0.089	1.588 ± 0.111	1 ± 0.219	1.469 ± 0.195
PE(18:0_20:4)	0.74 ± 0.086	1.038 ± 0.045	1.026 ± 0.231	1.259 ± 0.246

PS

	control	silymarin	control	silybin
PS(16:0_18:1)	0.116 ± 0.015	0.147 ± 0.01	0.109 ± 0.009	0.117 ± 0.009
PS(18:0_18:1)	0.132 ± 0.014	0.187 ± 0.008	0.672 ± 0.072	0.822 ± 0.091
PS(18:1_18:1)	0.806 ± 0.092	1.056 ± 0.053	0.158 ± 0.01	0.173 ± 0.014
PS(18:0_18:2)	0.09 ± 0.01	0.122 ± 0.009	0.103 ± 0.015	0.111 ± 0.011

PI

	control	silymarin	control	silybin
PI(16:0_18:1)	0.206 ± 0.013	0.316 ± 0.016	0.159 ± 0.015	0.18 ± 0.024
PI(18:1_18:1)	0.228 ± 0.014	0.383 ± 0.011	0.207 ± 0.03	0.252 ± 0.044
PI(16:0_20:4)	0.046 ± 0.004	0.059 ± 0.002	0.054 ± 0.021	0.039 ± 0.006
PI(18:0_20:4)	0.31 ± 0.034	0.415 ± 0.009	0.195 ± 0.027	0.193 ± 0.036

PG

	control	silymarin	control	silybin
PG(16:1_18:1)	0.118 ± 0.001	0.176 ± 0.015	0.148 ± 0.02	0.167 ± 0.024
PG(18:0_18:1)	0.133 ± 0.006	0.149 ± 0.006	0.228 ± 0.034	0.248 ± 0.053
PG(18:1_18:1)	0.055 ± 0.002	0.081 ± 0.001	0.075 ± 0.011	0.106 ± 0.022

SM

	control	silymarin	control	silybin
14:0 SM	0.02 ± 0.002	0.02 ± 0.003	0.027 ± 0.003	0.03 ± 0.001
16:0 SM	0.447 ± 0.035	0.65 ± 0.032	0.607 ± 0.061	0.661 ± 0.044
20:0 SM	0.492 ± 0.036	0.788 ± 0.036	0.6 ± 0.06	0.629 ± 0.029
22:0 SM	0.716 ± 0.055	1.3 ± 0.092	0.738 ± 0.035	0.909 ± 0.048

TG

	control	silymarin	control	silybin
16:0_16:0_16:1	0.666 ± 0.141	0.712 ± 0.14	0.735 ± 0.132	0.432 ± 0.049
16:0_16:1_16:1	0.461 ± 0.092	0.497 ± 0.08	0.244 ± 0.035	0.14 ± 0.006
16:0_16:0_18:1	1.532 ± 0.306	1.649 ± 0.317	1.515 ± 0.296	1.036 ± 0.155
16:0_18:0_18:1	0.907 ± 0.188	1.051 ± 0.209	0.917 ± 0.271	0.505 ± 0.115
16:0_16:1_18:0	0.297 ± 0.062	0.325 ± 0.072	0.345 ± 0.103	0.186 ± 0.041
16:0_16:1_18:2	0.12 ± 0.032	0.114 ± 0.025	0.148 ± 0.032	0.086 ± 0.006
16:0_16:1_18:1	1.153 ± 0.215	1.244 ± 0.212	1.312 ± 0.155	0.8 ± 0.028
16:1_16:1_18:0	0.093 ± 0.02	0.101 ± 0.02	0.116 ± 0.025	0.059 ± 0.006
16:1_16:1_18:1	0.294 ± 0.066	0.298 ± 0.058	0.389 ± 0.061	0.225 ± 0.013
16:0_18:1_18:2	0.387 ± 0.093	0.396 ± 0.085	1.375 ± 0.358	0.879 ± 0.236
16:1_18:1_18:2	0.203 ± 0.058	0.188 ± 0.045	0.281 ± 0.055	0.167 ± 0.009
16:0_16:1_20:3	0.141 ± 0.039	0.129 ± 0.03	0.191 ± 0.042	0.113 ± 0.008

50 100 200 [%]

Figure S5. Phospholipid and TG profiles of human monocytes and hepatocytes upon treatment with silymarin or silybin. (A, B) Human primary monocytes (A) and HepG2 cells (B) were treated as described in Figure 1. Heatmap showing the absolute abundance of phospholipid and TG species. Values are given as nmol / 1×10^6 cells for PC and units / 1×10^6 cells for PE, PS, PI, PG, SM and TG. Data are presented as mean ± SEM. The color indicates percentage changes relative to control. Data and the number of experiments are identical to Figure 1.

mouse liver

PC

	control	silybin
PC(16:0_16:0)	37.8 ± 2.2	53 ± 6.8
PC(16:0_18:1)	101.6 ± 6.5	106.2 ± 14.7
PC(18:0_18:1)	48.4 ± 2.9	50.9 ± 6.8
PC(18:1_18:1)	22.5 ± 1.4	14.9 ± 2.4
PC(16:0_18:2)	161.3 ± 12	251.4 ± 40
PC(18:0_18:2)	81.2 ± 4.9	124 ± 16.6
PC(16:0_20:3)	47.7 ± 2.5	43.9 ± 6.9
PC(16:0_20:4)	83 ± 10.5	105.1 ± 18.1
PC(18:0_20:4)	14.5 ± 1.2	13.1 ± 2.5
PC(18:1_20:1)	7.5 ± 0.6	7.2 ± 0.8
PC(18:1_20:4)	27.7 ± 1.9	45 ± 6.4
PC(32:0 E (16:0))	9.5 ± 0.9	10.5 ± 1.4
PC(34:2 E (18:2))	55.2 ± 4.3	75.5 ± 12.5

PE

	control	silybin
PE(16:0_18:1)	25.1 ± 2.8	20.7 ± 1.8
PE(18:0_18:1)	4.4 ± 0.3	4.38 ± 0.2
PE(18:1_18:1)	8.6 ± 0.6	5.8 ± 0.3
PE(16:0_18:2)	43.3 ± 2.6	61.3 ± 4.2
PE(18:0_18:2)	30.6 ± 1.8	43.3 ± 2.0
PE(18:1_18:2)	22.1 ± 1.3	22.5 ± 1.0
PE(16:0_20:3)	10.9 ± 0.7	10.57 ± 1.0
PE(16:0_20:4)	79.9 ± 4.5	89.9 ± 5.5
PE(18:0_20:4)	185.1 ± 11.5	237.4 ± 8.5

PS

	control	silybin
PS(16:0_20:4)	5.4 ± 0.3	5.5 ± 0.3
PS(18:0_20:4)	34.2 ± 1.9	43.4 ± 1.8
PS(18:0_22:6)	21.1 ± 1.3	26.9 ± 1.2

PI

	control	silybin
PI(16:0_20:3)	1.3 ± 0.1	1.1 ± 0.1
PI(16:0_20:4)	16.5 ± 0.5	18.1 ± 1.5
PI(18:0_20:4)	100.8 ± 2.3	124.1 ± 6.4

PG

	control	silybin
PG(16:0_18:1)	8.3 ± 0.2	7.9 ± 0.3
PG(18:1_18:1)	1 ± 0.1	1.0 ± 0.1
PG(16:0_18:2)	2.8 ± 0.1	3.3 ± 0.2
PG(18:1_18:2)	0.6 ± 0.0	0.8 ± 0.0

CE

	control	silybin
CE(16:0)	0.002 ± 0.000	0.003 ± 0
CE(18:0)	0.001 ± 0.000	0.001 ± 0.000
CE(16:1)	0.003 ± 0.000	0.003 ± 0.000
CE(18:1)	0.011 ± 0.001	0.013 ± 0.002
CE(18:2)	0.023 ± 0.002	0.03 ± 0.002
CE(18:3)	0.002 ± 0.000	0.002 ± 0.000
CE(20:4)	0.017 ± 0.000	0.033 ± 0.004
CE(22:6)	0.006 ± 0.000	0.015 ± 0.002

SM

	control	silybin
16:0 SM	2.2 ± 0.2	3.4 ± 0.2
20:0 SM	47.4 ± 4.9	59.9 ± 3.8
22:0 SM	20.7 ± 1.5	25.8 ± 1.9
22:1 SM	11.5 ± 0.9	9.1 ± 0.6
22:2 SM	27.7 ± 2.9	27.8 ± 1.7

TG

	control	silybin
16:0_16:0_16:0	0.099 ± 0.006	0.111 ± 0.006
16:0_16:0_16:1	0.196 ± 0.016	0.12 ± 0.005
16:0_16:0_18:1	1.068 ± 0.067	0.886 ± 0.054
16:0_16:1_16:1	0.117 ± 0.009	0.053 ± 0.008
16:0_16:1_18:1	0.782 ± 0.046	0.303 ± 0.024
16:0_18:1_18:1	5.118 ± 0.203	3.086 ± 0.163
16:0_16:1_18:0	0.025 ± 0.002	0.023 ± 0.002
16:1_16:1_18:1	0.088 ± 0.012	0.054 ± 0.012
16:0_16:0_18:2	1.614 ± 0.071	1.039 ± 0.107
16:0_16:1_18:2	0.562 ± 0.077	0.26 ± 0.059
16:0_18:0_18:2	3.419 ± 0.139	2.111 ± 0.107
16:0_18:1_18:2	3.364 ± 0.383	2.26 ± 0.376
16:1_18:1_18:2	0.305 ± 0.051	0.252 ± 0.064
18:0_18:1_18:2	0.31 ± 0.032	0.296 ± 0.033
16:0_18:2_18:2	1.797 ± 0.39	1.587 ± 0.387
16:0_16:1_20:3	0.199 ± 0.038	0.198 ± 0.056
16:0_18:0_20:3	0.216 ± 0.014	0.106 ± 0.009
16:0_16:0_20:4	1.273 ± 0.274	1.033 ± 0.237

50 100 150 [%]

Figure S6. Phospholipid, TG and CE profile of mouse liver upon gavage of silybin. Mice were treated as described in Figure 1. Heatmap showing the absolute abundance of phospholipid, TG and CE species. Values are given as nmol for PC and units for PE, PS, PI, PG, SM and TG. Data are presented as mean ± SEM. The color indicates percentage changes relative to control. Data and the number of experiments are identical to Figure 1.

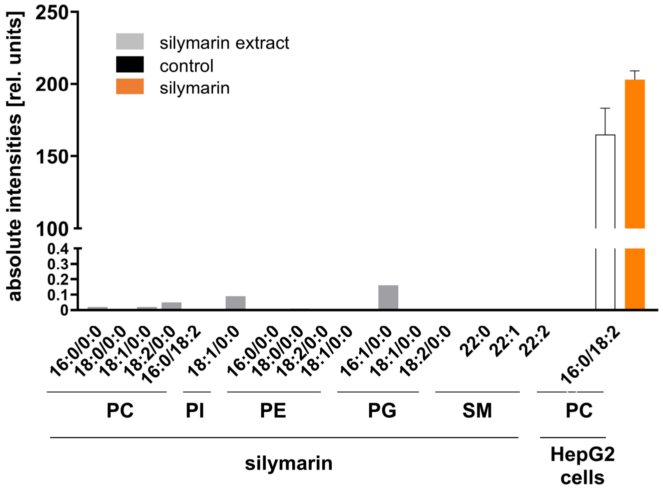

Figure S7. (Lyso)phospholipid content of silymarin in comparison to hepatocytes. Absolute abundance of (lyso)phospholipids in 10 µg silymarin or 3×10^5 HepG2 cells, which corresponds to the treatment of hepatocytes with 10 µg/ml under our experimental conditions. Lipid analysis of HepG2 cells focused on those species from Figure S5 and S6 that are present in silymarin, i.e., PC(16:0/18:2). Single value or mean + SEM; n = 1 (silymarin) or n = 3 (HepG2 cells).

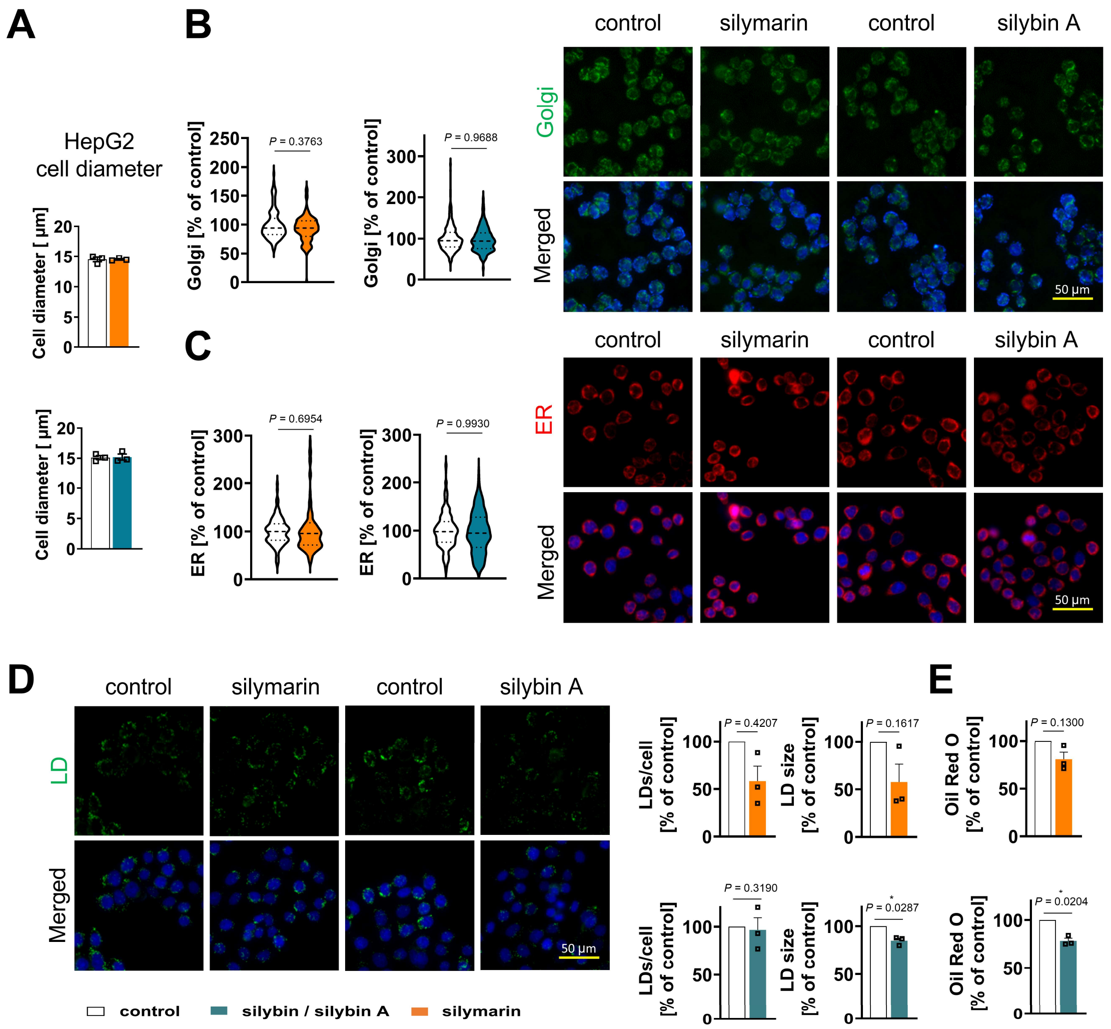

Figure S8. Impact of silymarin and silybin A on intracellular membrane compartments and lipid droplets. HepG2 cells were incubated with silymarin (10 µg/ml), silybin A (20 µM) or vehicle control (ethanol for silymarin, DMSO for silybin A) for 24 h. (A) Cell diameter of HepG2 cells determined with a Vi-Cell™ XR cell counting system. Individual values and means + SEM; n = 3. (B-D) Labeling of Golgi with CellLight™ Golgi-GFP (B), ER with ER-Tracker™ Red (BODIPY™ TR Glibenclamide) (C) and lipid droplets with BioTracker™ 488 Green Lipid Dye Biotracker (D); nuclei were stained with Hoechst 33342 (blue). Violine plots show quantification of the mean intensities for 130 control cells (ethanol), 122 silymarin-treated cells, 551 control cells (DMSO), and 335 silybin A-treated cells (B); and for 127 control cells (ethanol), 131 silymarin-treated cells, 404 control cells (DMSO), and 366 silybin A-treated cells (C) from n = 3 independent experiments. Median and interquartile range are indicated as doted lines. Quantification of the number and size of lipid droplets using the "Analyze Particles" tool in ImageJ for 103 control cells (ethanol), 126 silymarin-treated cells, 105 control cells (DMSO), and 120 silybin A-treated cells (D) from n = 3 independent experiments. The statistics were performed on the mean values of n = 3 independent experiments. (E) Relative lipid droplet content analyzed by Oil Red O staining. Individual values and mean + SEM; n = 3. *$P < 0.05$ vs. vehicle control. Two-tailed paired Student's *t*-test on raw data (A, B, C, E). Scale bar 50 µm.

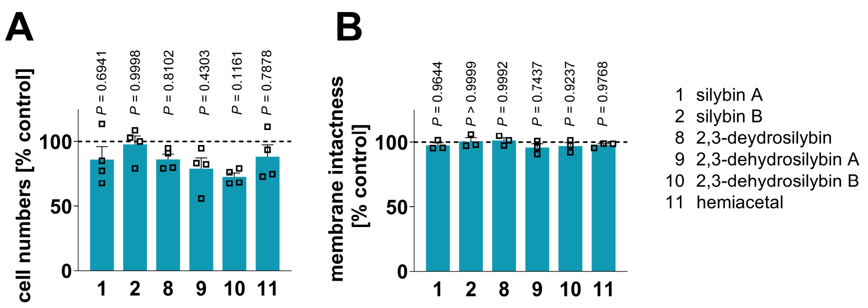

Figure S9. Effect of silybin derivatives on cell number and cell membrane intactness. HepG2 cells were treated with the indicated compounds (20 µM) or vehicle (DMSO) for 24 h. (A) Cell numbers; (B) membrane intactness measured by trypan blue staining. Individual values and mean + SEM; n = 3 (B) or n = 4 (A). *P* values vs. vehicle control; repeated measures one-way ANOVA + Tukey HSD *post hoc* tests.

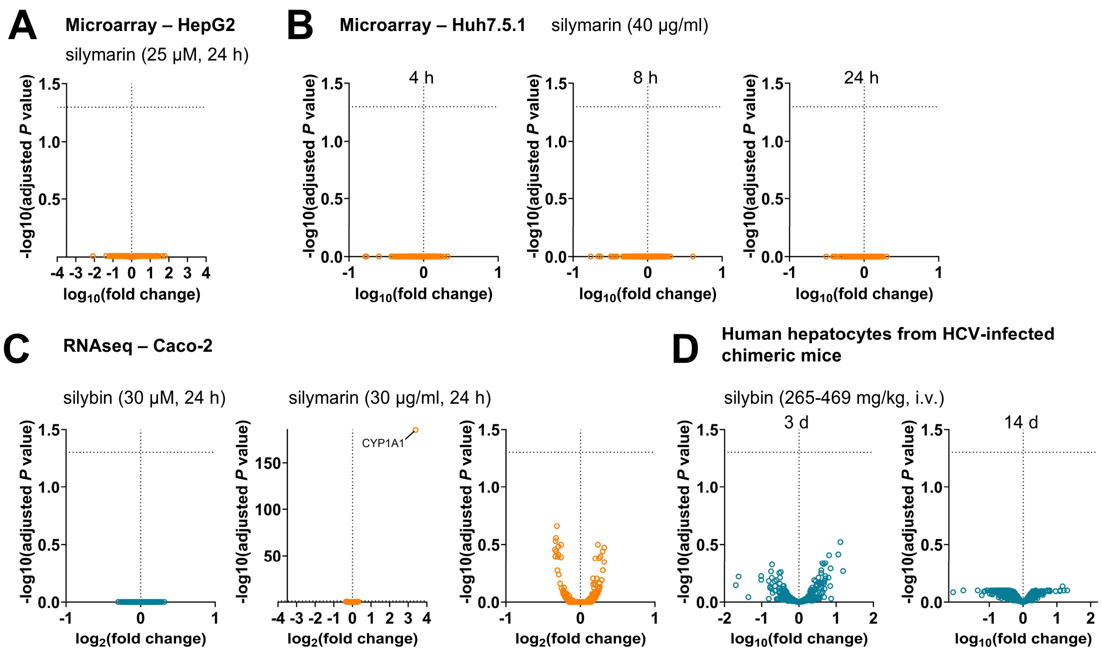

Figure S10. Volcano plots showing the data from Fig. 6A-D adjusted for multiple comparisons. Statistical calculations were performed by pairwise comparison of treatment and control groups using the GEO2R interactive webtool (https://www.ncbi.nlm.nih.gov/geo/geo2r/)[1]. Adjusted *P* values were calculated by multiple *t*-tests, with correction for multiple comparisons according to Benjamini and Hochberg (false discovery rate 5%) and autodetection for log-transformation. The dashed line indicates an adjusted *P* value of 0.05.

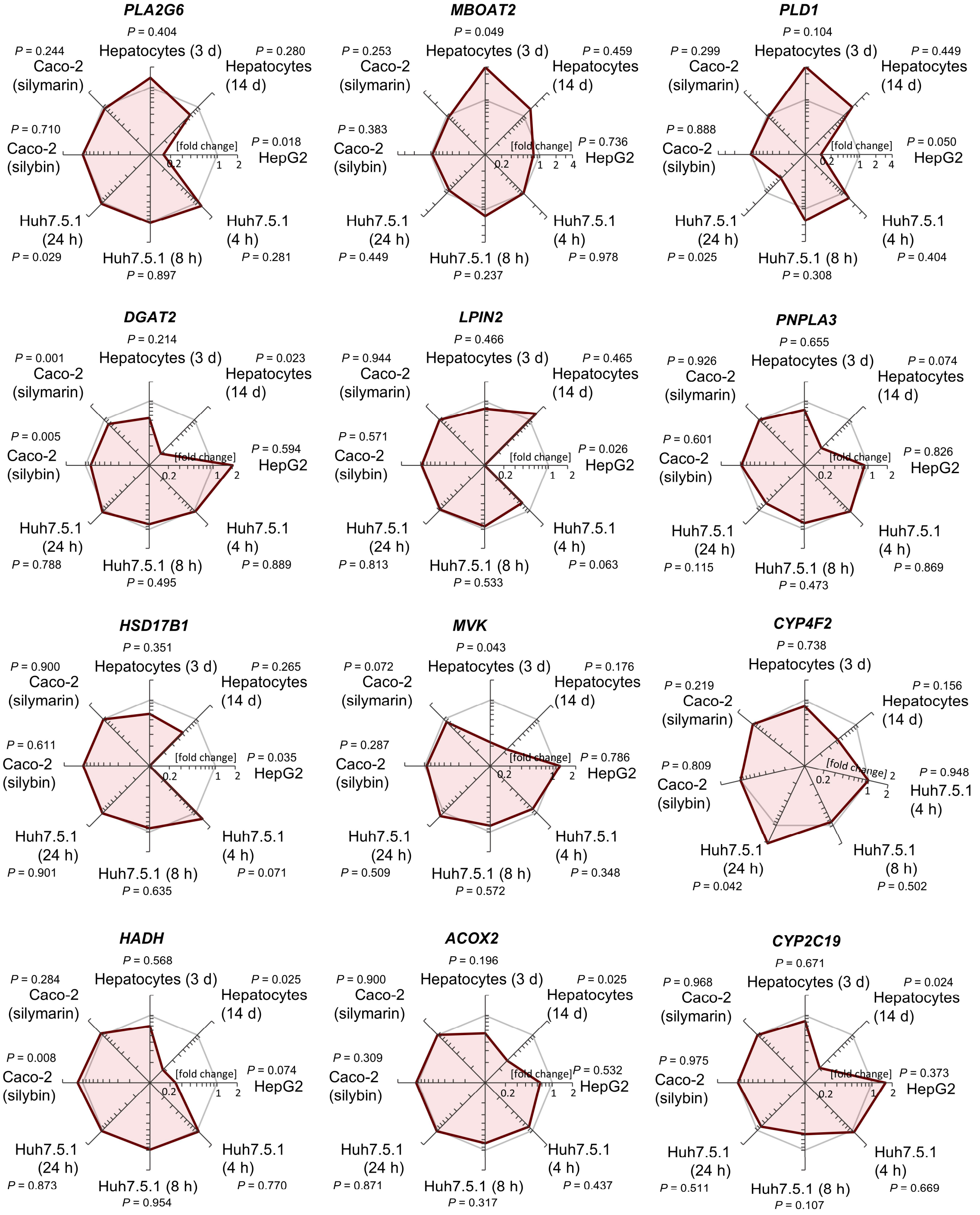

Figure S11. Lipid metabolic enzymes differentially regulated by silymarin/silybin. Comparative analysis of transcriptome data from silymarin-treated HepG2 and Huh7.5.1 hepatocarcinoma cells, silybin-and silymarin-treated CaCo-2 colon carcinoma cells, and hepatocytes derived from HCV-infected mice receiving silybin. Radar plots indicating the fold change in *PLA2G6*, *MBOAT2, PLD1, DGAT2, LPIN2, PNPLA3, HSH17B1, MVK, CYP4F2, HADH, ACOX2,* and *CYP2C19* expression by silymarin (HepG2, Huh7.5.1, CaCo-2) or silybin (hepatocytes, CaCo-2) relative to vehicle control. Non-adjusted *P* values given vs. vehicle control; multiple two-tailed unpaired Student's *t*-tests. Data are identical to Figure 4.

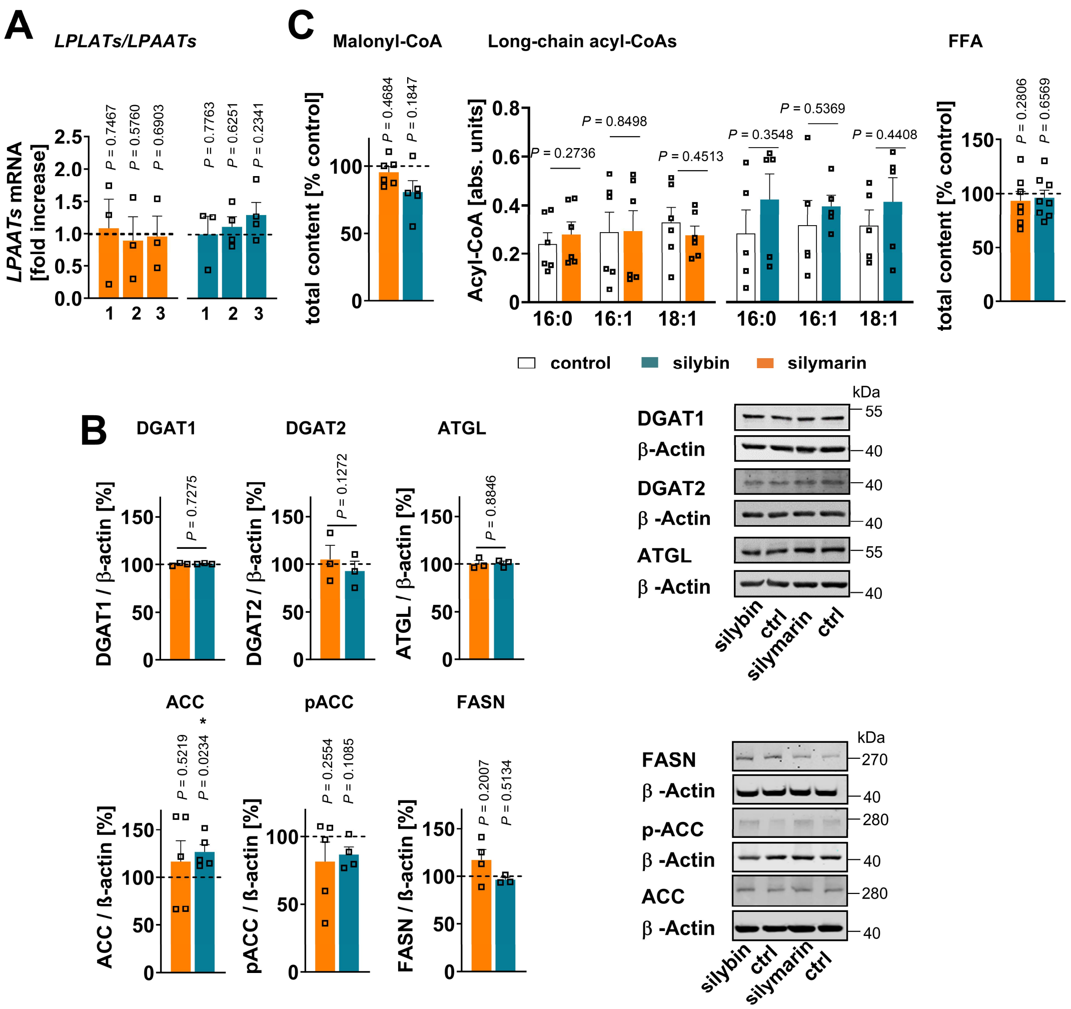

Figure S12. Remodeling of *de novo* phospholipid biosynthesis and TG metabolism. (A-C) HepG2 cells were incubated with silymarin (10 µg/ml), silybin (20 µM) or vehicle (ethanol for silymarin, DMSO for silybin) for 24 h; (A) mRNA levels of *LPLAT/LPAAT1-3* normalized to β-actin (*LPLATs/LPAATs* silybin) or *GAPDH* (*LPLATs/LPAATs* silymarin). Individual values and mean + SEM as fold-change of control; n = 3 (*LPLATs/LPAATs* silymarin, *LPLAT1/LPAAT1* silybin) and n = 4 (*LPLATs/LPAATs* silybin except *LPLAT1/LPAAT1*). (B) Protein expression of DGAT1, DGAT2, ATGL/PNPLA2, ACC1/2 and FASN, phosphorylation of ACC1/2. Individual values and mean + SEM; n = 3 (DGAT1, DGAT2, ATGL, FASN silybin), n = 4 (pACC silybin, FASN silymarin), and n = 5 (ACC, pACC silymarin). Individual values and mean + SEM; n = 3. Representative Western blots are shown. (C) Effects of silymarin and silybin on the cellular content of malonyl-CoA, long-chain acyl-CoAs (normalized to the internal standard [$^{13}C_3$]-malonyl-CoA) and FFAs. Individual values and mean + SEM; n = 5 (silybin, malonyl-CoA and long-chain acyl-CoAs) and n = 6 (silymarin, malonyl-CoA and long-chain acyl-CoAs), n = 7 (silymarin, FFA) or n = 8 (silybin, FFA). *$P < 0.05$ vs. vehicle controls; two-tailed paired Student's t-tests.

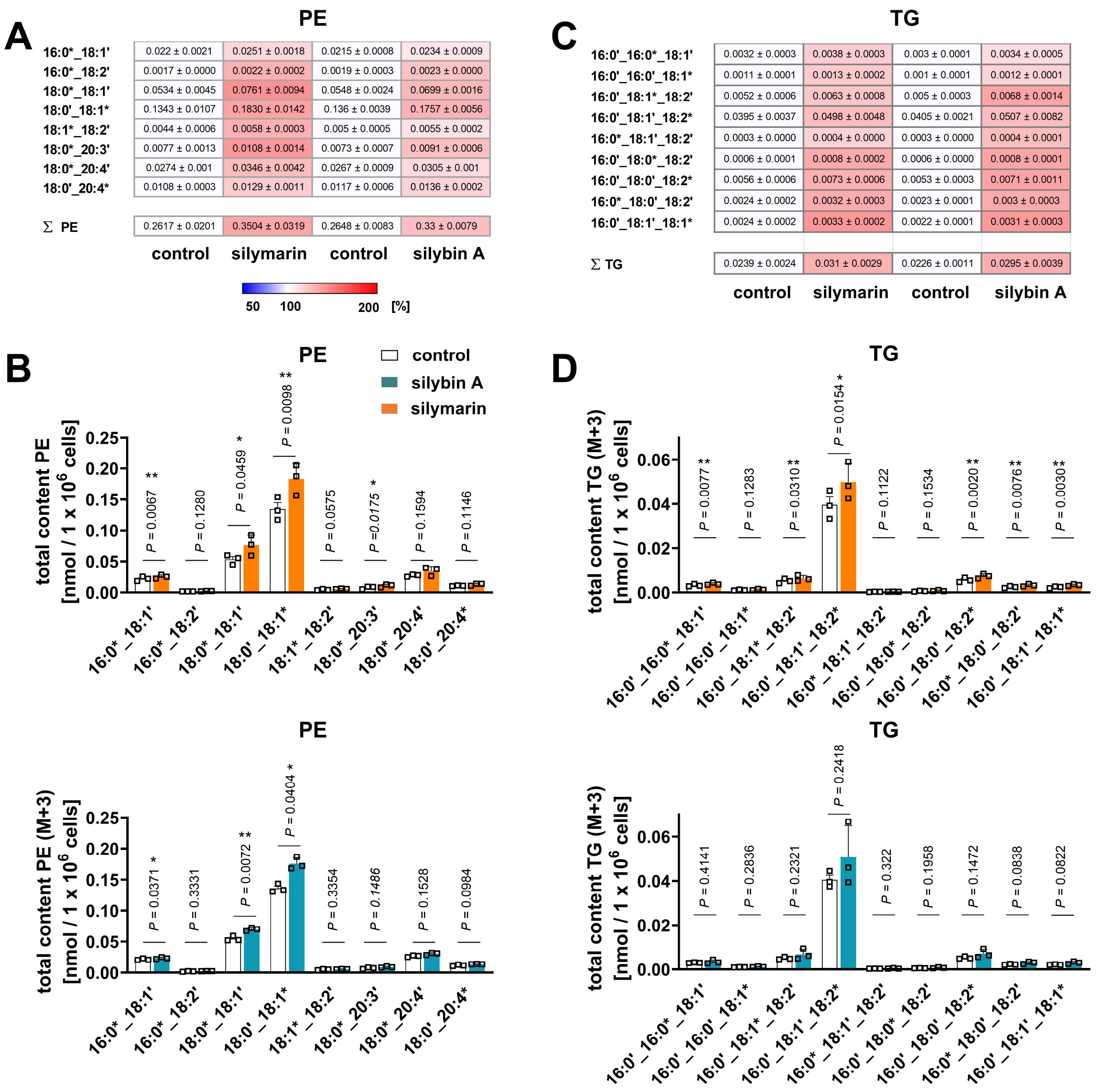

Figure S13. Incorporation of sodium acetate-$^{13}C_2$, d_3 into phospholipid and TG species of human HepG2 cells treated with silymarin or silybin A. (A-D) Human HepG2 cells were treated as described in Figure 4I and J. (A, B) The heatmap and bar charts display the absolute amount of isotopically-labeled PE species, corrected for naturally occurring isotopes and normalized to internal standard and cell number in nmol / 1×10^6 cells, as mean ± SEM, with color indicating percentage changes to the vehicle control. Data and the number of experiments are identical to Figure 4I. (C, D) The heatmap and bar charts display the absolute amounts of isotopically labeled TG species, corrected for naturally occurring isotopes and normalized to the internal standard and cell count in nmol / 1×10^6 cells. Data are presented as mean ± SEM. The colors represent percentage changes relative to the vehicle control. Data and the number of experiments are identical to Figure 4J. Fatty acids that incorporated isotopically labeled sodium acetate (M+3) are indicated with asterisks (*) and fatty acids that remained in the molecule after fragmentation are indicated with primes ('). (B, D) Individual values and means + SEM; n = 3. *$P < 0.05$, *$P < 0.001$ vs. vehicle controls; two-tailed paired Student's t-tests.

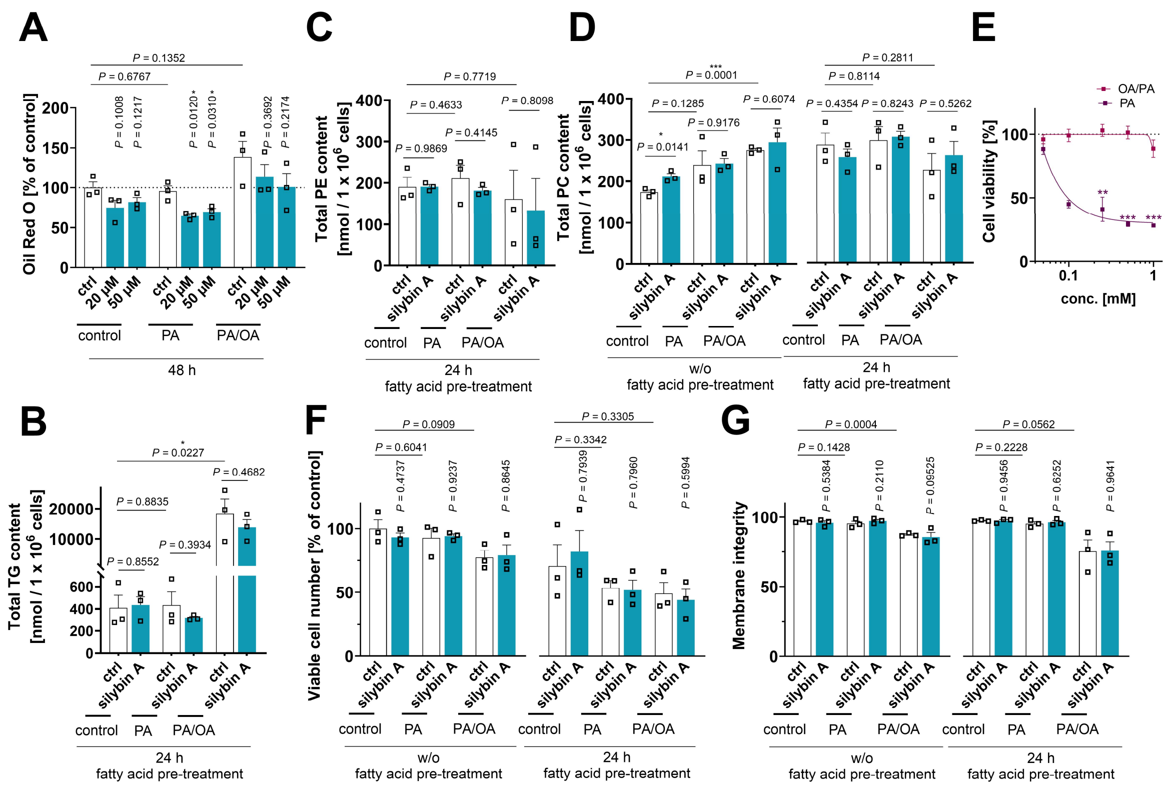

Figure S14. Comparison of the silybin A effects in non-challenged hepatocytes and cell-based disease models of MAFLD and acute lipotoxicity. (A) HepaRG cells were treated with 0.1 mM palmitate (PA) or a mixture of PA/oleate (OA) in a 1:2 ratio (in total 1 mM) together with vehicle (DMSO, 0.5%) or compounds for 48 h. Relative lipid droplet content was determined by Oil Red O staining. (B-D) HepaRG cells were either pre-treated with 0.1 mM palmitate (PA) or a mixture of PA/oleate (OA) in a 1:2 ratio (in total 1 mM) for 24 h followed by vehicle (DMSO, 0.5%) or silybin A (20 µM) treatment or cells were directly co-treated with vehicle (DMSO, 0.5%) or silybin A (20 µM), and the incubation was prolonged for a further 24 h. Total levels of TG (B), PE (C), and PC (D) determined by UPLC-MS/MS. (E) Cell viability measured by MTT assay. (F) Viable cell numbers. (G) Cell membrane integrity determined by trypan blue staining. Individual values and mean + SEM or ± SEM, n = 2 (E, 0.1 mM PA) or n = 3 (A-G, except D, 0.1 mM PA). *P < 0.05, ***P < 0.001 vs. control; two-tailed unpaired Student's t-test.

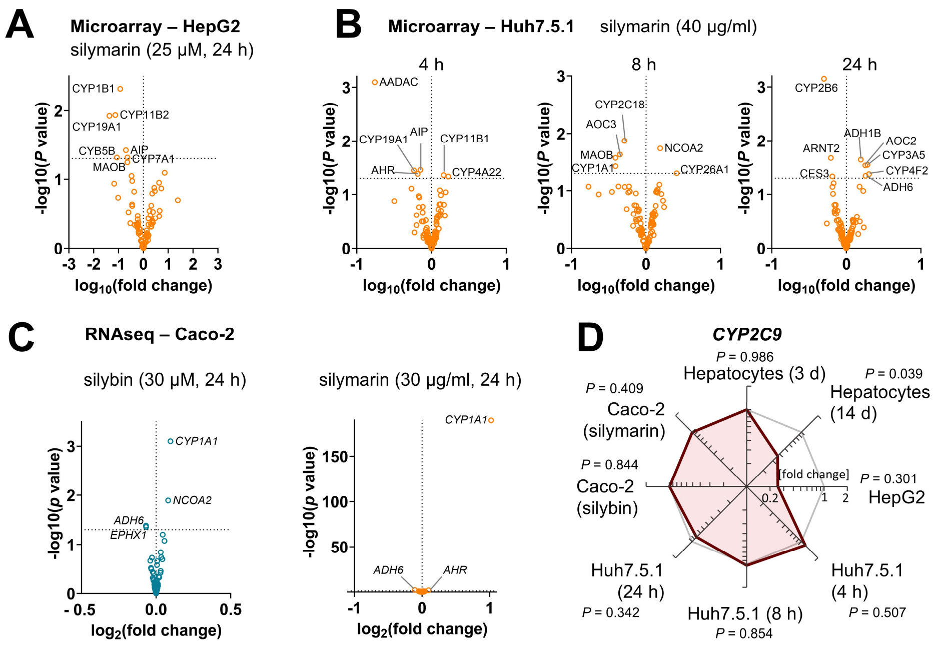

Figure S15. Silymarin/silybin kinetically controls the mRNA levels of genes involved in drug metabolism. Comparative analysis of transcriptome data from silymarin-treated HepG2 and Huh7.5.1 hepatocarcinoma cells, silybin- and silymarin-treated CaCo-2 colon carcinoma cells, and hepatocytes derived from HCV-infected mice receiving silybin. (A-C) Volcano plots compare the expression of proteins involved in drug metabolism upon silymarin (A-C) or silybin (C) treatment vs. vehicle control. Statistical calculations were performed by pairwise comparison of treatment and control groups using the GEO2R interactive webtool (https://www.ncbi.nlm.nih.gov/geo/geo2r/)[1]. *P*-values were calculated by multiple *t*-tests without correction for multiple comparisons. The dashed line indicates a *P*-value of 0.05. (D) Radar plots indicating the fold change in *CYP2C9* by silymarin (HepG2, Huh7.5.1, CaCo-2) or silybin (hepatocytes, CaCo-2) relative to vehicle control. Non-adjusted *P* values are given vs. vehicle control; multiple two-tailed unpaired Student's *t*-tests.

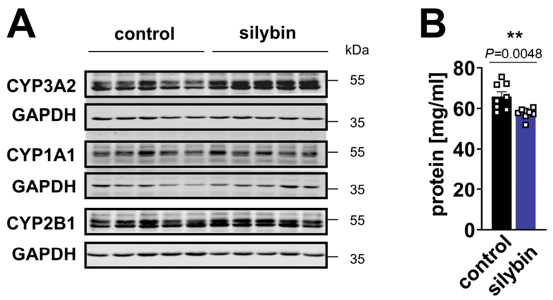

Figure S16. Impact of silybin on CYP expression in mouse liver. (A) Western blots for the densitometric data shown in Figure 8C are representative of n = 5 mice/group; (B) Protein concentration of the 9,000×g supernatant of the liver homogenate. Individual values and mean + SEM; n = 8 mice/group. $^{**}P < 0.01$ vs. vehicle control; two-tailed unpaired Student's *t*-test.

Supplementary Notes

Supplementary Note 1: Cholesterol and CE metabolism

The effects of silybin/silymarin on CEs, the second major neutral lipid in lipid droplets after TGs, are less consistent. In some settings, silybin/silymarin stimulates cholesterol biosynthesis by upregulating the transcription factor *SREBP1* (Figure 4A) and repressing its endoplasmic reticulum anchor *INSIG1* (Figure 4D and E), thereby favoring the transfer of *SREBP1* to the Golgi for proteolytic procession to the mature form[2-4]. On the contrary, several SREBP-regulated target genes were downregulated (*ACACA, ELOVL6, MVK*, Figure 4A, E, Figure S11), except for FASN, which was upregulated as expected (Figure 4C). In other settings, silybin/silymarin reduces the expression of cholesterol biosynthetic enzymes (*MVK, TM7SF2*, Figure 4C, D, and E, and Figure 11). Such counter-regulation could be explained by initially increased levels of sterols, which then bind to the cholesterol sensor *INSIG1* (Figure S6 D and E) and suppress SREBP1 signaling (along with target protein expression) without necessarily interfering with SREBP1 expression[2]. In addition to canonical cholesterol biosynthesis, increased lipoprotein and sterol uptake (*LRP2*) (Figure 4B and C) and possibly endosomal cholesterol transport (*STARD3NL*) (Figure 4C) may further add to the accumulation of intracellular CE. In strong support of this hypothesis, silymarin administration to mice substantially elevated the hepatic CE content (Figure 1D). However, the increase in esterified cholesterol levels did not seem to be translated into enhanced cholesterol metabolism, as the expression of various sterol-metabolizing enzymes (*CYP1A1, CYP1B1, CYP2C8, CYP3A4, CYP7A1, CYP11B2, CYP17A1, CYP19A1, CYP27A1, ABCB11, SLC10A1, SLC27A5, HSD3B2, HSD17B1, HSD17B3, AKR1D1, AKR1C3, EBP, BAAT, STS*) is largely repressed (Figure 4A, B, C, D and E, and Figure S11), with a few exceptions (*CYP1A1, CYP1B1, CYP11B1, AKR1C3*, Figure 4B-D). Together, silymarin/silybin consistently downregulate anabolic and catabolic sterol metabolism, while exerting complex, partially opposite effects on cholesterol biosynthesis.

Supplementary Note 2: Vitamin A metabolism

In addition, silymarin/silybin differentially regulates a significant number of genes related to vitamin A metabolism, including retinoic acid biosynthesis (*CYP1B1, CYP3A4, ADH1B, ADH6,* Figure 4A, Figure S14A-C), degradation (*CYP2C18, CYP3A5, CYP26A1, CYP2C8*, Figure 8A and B, Figure S14B), and vitamin A storage (*DGAT1, PNPLA3 and ATGL/ PNPLA2,* Figure 4A, B, E, and F, Figure S11).

Vitamin A is stored as retinyl esters in lipid droplets in the liver and shares common metabolic pathways with TG. This involves genetic risk factors for MAFLD, such as *DGAT1, PNPLA3*, and *ATGL/PNPLA2*, which participate in retinol ester synthesis and degradation[5,6]. Vitamin A plays a central role in the regulation of hepatic lipid metabolism, including lipogenesis, lipid transport, and lipid catabolism[6], and disturbed vitamin A metabolism has been associated with MAFLD[5,6]. It is therefore remarkable that our comparative transcriptome analysis revealed that numerous genes involved in vitamin A metabolism (including *CYP1B1, CYP3A4, ADH1B, ADH6, CYP2C18, CYP3A5, CYP26A1, CYP2C8, DGAT1, PNPLA2/ATGL* and possibly *PNPLA3*) are subject to regulation by silybin/silymarin. Further studies are needed to explore whether the interference with vitamin A metabolism by silymarin/silybin affects hepatic lipid metabolism and influences the development and/or progression of MAFLD.

References

1 Barrett, T. *et al.* NCBI GEO: archive for functional genomics data sets--update. *Nucleic Acids Res* **41**, D991-995, doi:10.1093/nar/gks1193 (2013).

2 Yang, T. *et al.* Crucial step in cholesterol homeostasis: sterols promote binding of SCAP to INSIG-1, a membrane protein that facilitates retention of SREBPs in ER. *Cell* **110**, 489-500, doi:10.1016/s0092-8674(02)00872-3 (2002).

3 Engelking, L. J., Cantoria, M. J., Xu, Y. & Liang, G. Developmental and extrahepatic physiological functions of SREBP pathway genes in mice. *Semin Cell Dev Biol* **81**, 98-109, doi:10.1016/j.semcdb.2017.07.011 (2018).

4 Chen, Y. *et al.* Maturation and activity of sterol regulatory element binding protein 1 is inhibited by acyl-CoA binding domain containing 3. *PLoS One* **7**, e49906, doi:10.1371/journal.pone.0049906 (2012).

5 Saeed, A. *et al.* Impaired Hepatic Vitamin A Metabolism in NAFLD Mice Leading to Vitamin A Accumulation in Hepatocytes. *Cell Mol Gastroenterol Hepatol* **11**, 309-325 e303, doi:10.1016/j.jcmgh.2020.07.006 (2021).

6 Saeed, A., Dullaart, R. P. F., Schreuder, T., Blokzijl, H. & Faber, K. N. Disturbed Vitamin A Metabolism in Non-Alcoholic Fatty Liver Disease (NAFLD). *Nutrients* **10**, doi:10.3390/nu10010029 (2017).

Uncropped WB from Figure 4

B

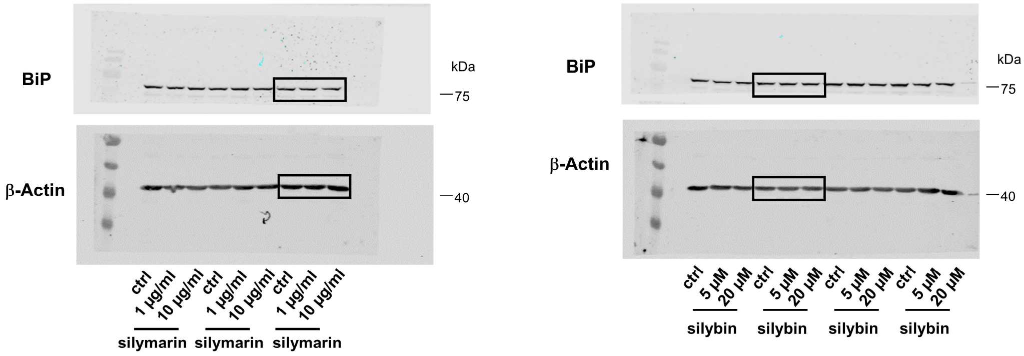

C

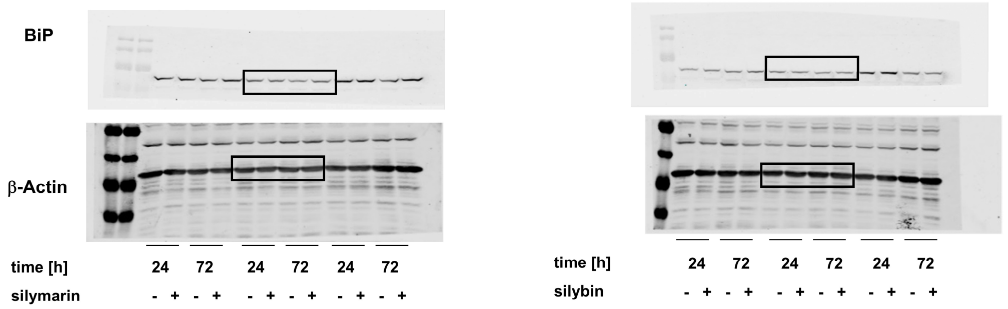

D

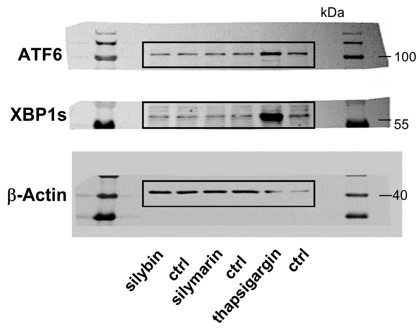

Uncropped WB from Figure S12

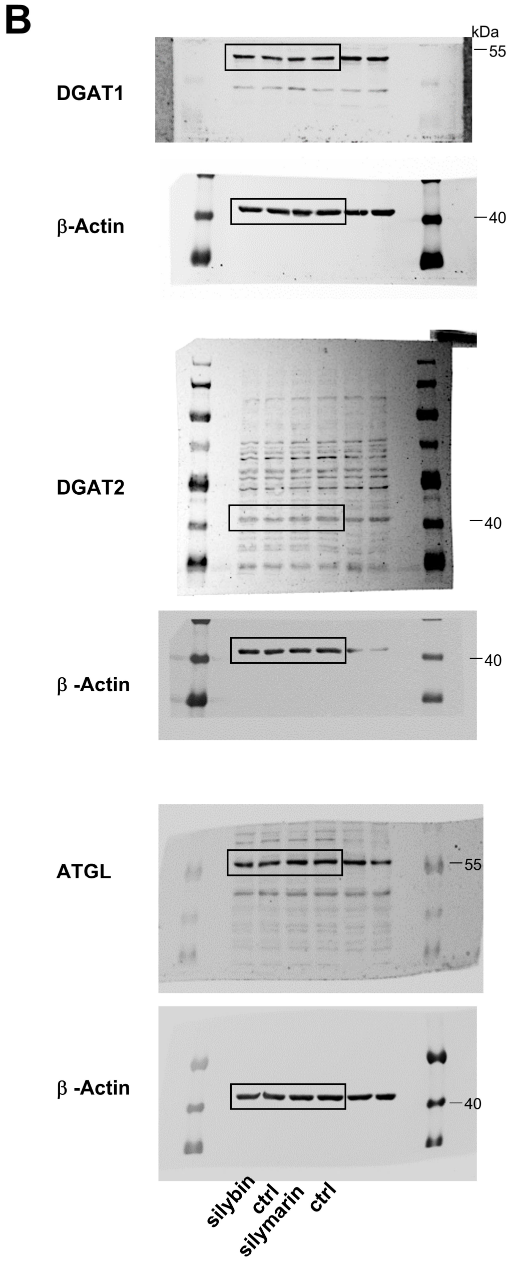

Uncropped WB from Figure S12

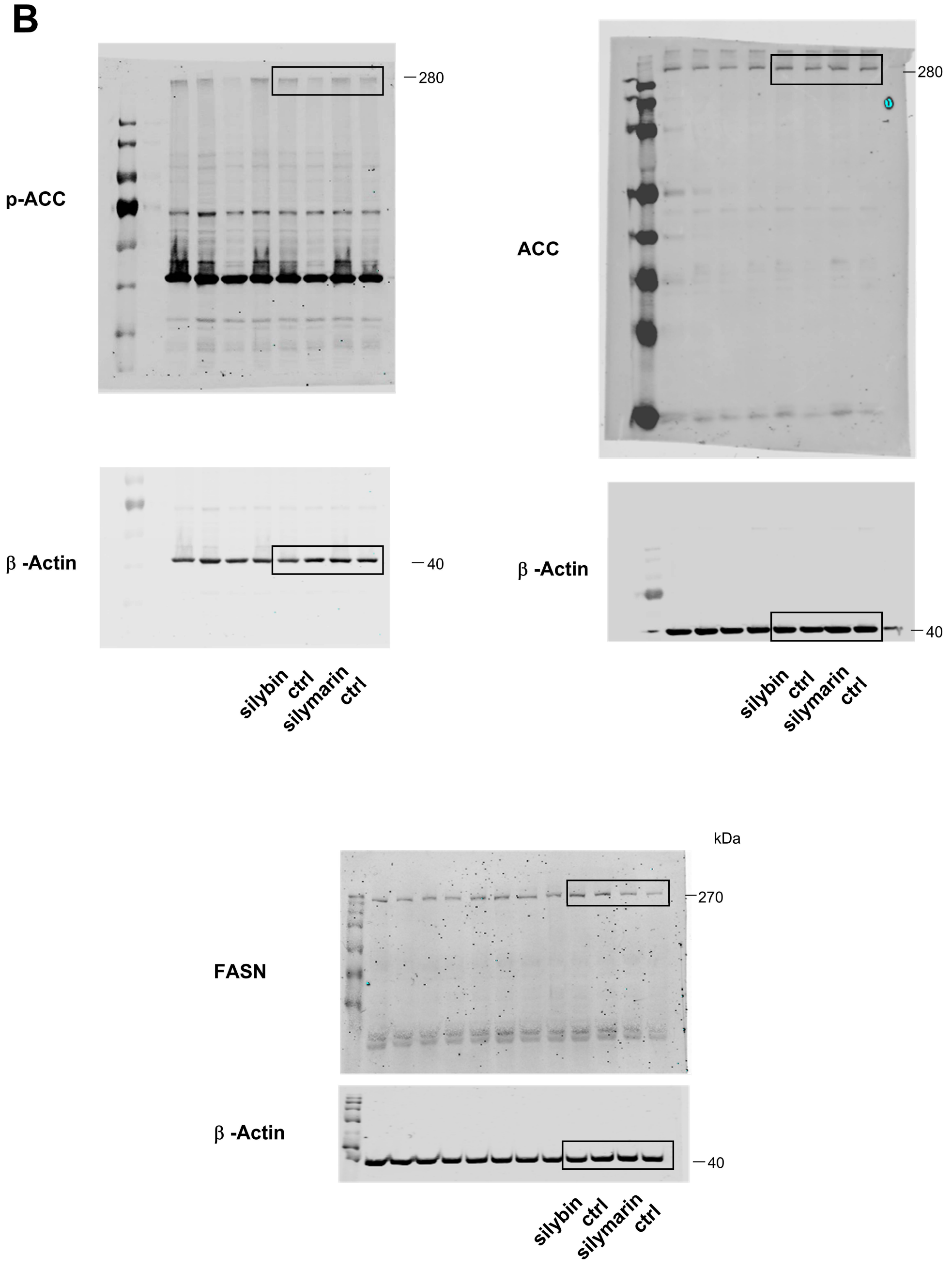

Uncropped WB from Figure S15

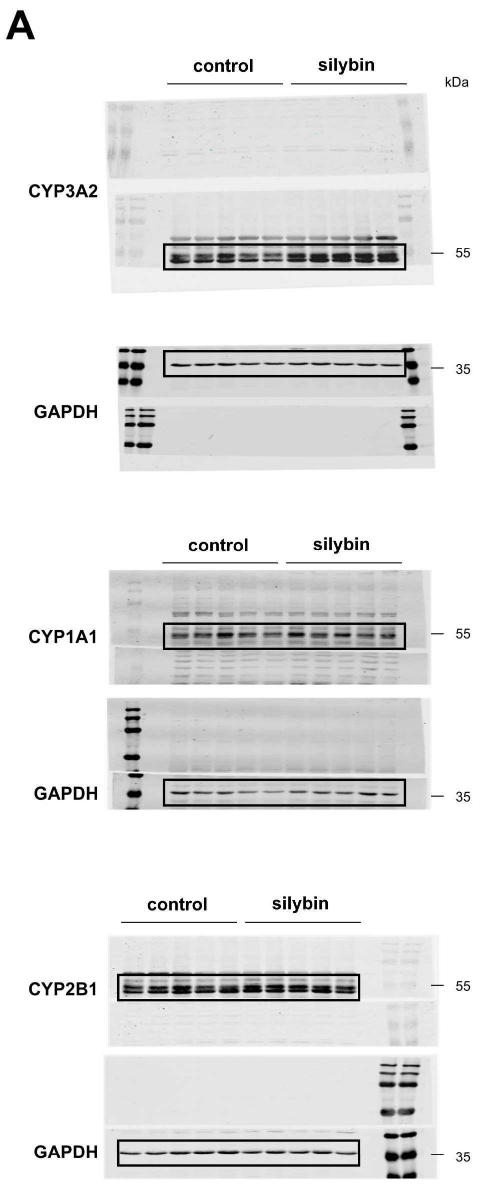

Supplement: Supplementary file 1 — Supplementary figures and notes. [file thnov15p2006s1.pdf]
